# Ischemic Stroke Is Associated with the *ABO* Locus: The EuroCLOT Study

**DOI:** 10.1002/ana.23838

**Published:** 2013-02-04

**Authors:** Frances M K Williams, Angela M Carter, Pirro G Hysi, Gabriela Surdulescu, Dylan Hodgkiss, Nicole Soranzo, Matthew Traylor, Steve Bevan, Martin Dichgans, Peter M W Rothwell, Cathie Sudlow, Martin Farrall, Kaisa Silander, Mari Kaunisto, Peter Wagner, Olli Saarela, Kari Kuulasmaa, Jarmo Virtamo, Veikko Salomaa, Philippe Amouyel, Dominique Arveiler, Jean Ferrieres, Per-Gunnar Wiklund, M Arfan Ikram, Albert Hofman, Giorgio B Boncoraglio, Eugenio A Parati, Anna Helgadottir, Solveig Gretarsdottir, Unnur Thorsteinsdottir, Gudmar Thorleifsson, Kari Stefansson, Sudha Seshadri, Anita DeStefano, Andreas Gschwendtner, Bruce Psaty, Will Longstreth, Braxton D Mitchell, Yu-Ching Cheng, Robert Clarke, Marco Ferrario, Joshua C Bis, Christopher Levi, John Attia, Elizabeth G Holliday, Rodney J Scott, Myriam Fornage, Pankaj Sharma, Karen L Furie, Jonathan Rosand, Mike Nalls, James Meschia, Thomas H Mosely, Alun Evans, Aarno Palotie, Hugh S Markus, Peter J Grant, Tim D Spector, on Behalf of the EuroCLOT Investigators, the Wellcome Trust Case Control Consortium 2, MOnica Risk, Genetics, Archiving and Monograph, MetaStroke, and the International Stroke Genetics Consortium

**Affiliations:** 1 Department of Twin Research and Genetic Epidemiology, King's College LondonLondon, United Kingdom; 2 Division of Cardiovascular and Diabetes Research, University of LeedsLeeds, United Kingdom; 3 Wellcome Trust Sanger Institute, Wellcome Trust Genome CampusHinxton, United Kingdom; 4 Program in Medical and Population Genetics and Genetic Analysis Platform, Broad Institute of Massachusetts Institute of Technology and HarvardCambridge, MA; 5 Department of Medical Genetics, University of Helsinki and University Central HospitalHelsinki, Finland; 6 Stroke and Dementia Research Centre, St George's University of LondonLondon, United Kingdom; 7 Institute for Stroke and Dementia Research, Klinikum der Universität Munich, Ludwig-Maximilians-UniversityMunich, Germany; 8 Stroke Prevention Research Unit, University Department of Clinical Neurology, Oxford UniversityOxford, United Kingdom; 9 Division of Clinical Neurosciences, University of EdinburghEdinburgh, United Kingdom; 10 Wellcome Trust Centre for Human Genetics and Department of Cardiovascular Medicine, University of OxfordOxford, United Kingdom; 11 Institute for Molecular Medicine Finland, University of HelsinkiHelsinki, Finland; 12 Chronic Disease Epidemiology and Prevention Unit, National Institute for Health and WelfareHelsinki, Finland; 13 Department of Epidemiology and Public Health, Pasteur Institute of LilleLille, France; 14 Department of Epidemiology and Public Health, University of StrasbourgStrasbourg, France; 15 Department of Epidemiology, Faculty of MedicineToulouse-Purpan, Toulouse, France; 16 Department of Internal Medicine, University of UmeåUmeå, Sweden; 17 Department of Epidemiology, Erasmus University Medical CenterRotterdam, the Netherlands; 18 Department of Neurology, Research Hospital of the Neurological Institute “Carlo Besta,”Milan, Italy; 19 DeCODE GeneticsReykjavik, Iceland; 20 Boston University Schools of Medicine and Public HealthBoston, MA; 21 Framingham Heart StudyFramingham, MA; 22 Departments of Health Services, University of WashingtonSeattlem WA; 23 Epidemiology, Health Services, University of WashingtonSeattle, WA; 24 Medicine, and Health Services, University of WashingtonSeattle, WA; 25 Group Health Research Institute, Group HealthSeattle, WA; 26 Department of Neurology, University of WashingtonWA; 27 Department of Medicine, University of MarylandBaltimore, MD; 28 Clinical Trial Service Unit, University of OxfordOxford, United Kingdom; 29 University of InsubriaVarese, Italy; 30 Cardiovascular Health Research Unit, Department of Medicine, University of WashingtonSeattle, WA; 31 University of NewcastleCallaghan, Australia; 32 John Hunter HospitalNew Lambton Heights, Australia; 33 Hunter Medical Research InstituteNew Lambton, Australia; 34 Human Genetics Center and Institute of Molecular Medicine, University of Texas Health Sciences CenterHouston, TX; 35 Imperial College Cerebrovascular Research Unit, Imperial College LondonLondon, United Kingdom; 36 Center for Human Genetic Research, Massachusetts General Hospital and Harvard Medical SchoolBoston, MA; 37 Department of Neurology, Mayo ClinicJacksonville, FL, USA; 38 University of Mississippi Medical CenterJackson, MS; 39 Queen's University of BelfastBelfast, United Kingdom

## Abstract

**Objective:**

End-stage coagulation and the structure/function of fibrin are implicated in the pathogenesis of ischemic stroke. We explored whether genetic variants associated with end-stage coagulation in healthy REFVIDunteers account for the genetic predisposition to ischemic stroke and examined their influence on stroke subtype.

**Methods:**

Common genetic variants identified through genome-wide association studies of coagulation factors and fibrin structure/function in healthy twins (n = 2,100, Stage 1) were examined in ischemic stroke (n = 4,200 cases) using 2 independent samples of European ancestry (Stage 2). A third clinical collection having stroke subtyping (total 8,900 cases, 55,000 controls) was used for replication (Stage 3).

**Results:**

Stage 1 identified 524 single nucleotide polymorphisms (SNPs) from 23 linkage disequilibrium blocks having significant association (*p* < 5 × 10^–8^) with 1 or more coagulation/fibrin phenotypes. The most striking associations included SNP rs5985 with factor XIII activity (*p* = 2.6 × 10^–186^), rs10665 with FVII (*p* = 2.4 × 10^–47^), and rs505922 in the *ABO* gene with both von Willebrand factor (*p* = 4.7 × 10^–57^) and factor VIII (*p* = 1.2 × 10^–36^). In Stage 2, the 23 independent SNPs were examined in stroke cases/noncases using MOnica Risk, Genetics, Archiving and Monograph (MORGAM) and Wellcome Trust Case Control Consortium 2 collections. SNP rs505922 was nominally associated with ischemic stroke (odds ratio = 0.94, 95% confidence interval = 0.88–0.99, *p* = 0.023). Independent replication in Meta-Stroke confirmed the rs505922 association with stroke, beta (standard error, SE) = 0.066 (0.02), *p* = 0.001, a finding specific to large-vessel and cardioembolic stroke (*p* = 0.001 and *p* = < 0.001, respectively) but not seen with small-vessel stroke (*p* = 0.811).

**Interpretation:**

*ABO* gene variants are associated with large-vessel and cardioembolic stroke but not small-vessel disease. This work sheds light on the different pathogenic mechanisms underpinning stroke subtype. Ann Neurol 2013

Ischemic stroke is among the leading causes of death and disability in high-income countries.^1^ EuroCLOT is a European Union–funded multicenter study established to identify the genetic variants contributing to end-stage coagulation, as a means of exploring whether the same variants contribute to risk of ischemic stroke. It is known that genetic factors account for approximately 60% of the risk of thrombosis,^2^ and studies have demonstrated the influence of genetic factors on the individual components of coagulation and fibrinolysis. Furthermore, ex vivo measures of fibrin structure and fibrinolysis have been shown to be heritable.^3^ The nature of the structure and function of fibrin has been shown to influence clot behavior, and earlier work by the EuroCLOT consortium has demonstrated heritability of fibrin clot phenotypes measured by a high-throughput turbidimetric assay and several regions of linkage.^4^ The goal of this study was to extend these observations by using the genome-wide association (GWA) approach to identify common genetic loci associated with coagulation phenotypes and to determine whether associated loci were further associated with the clinically important phenotype ischemic stroke and its different subtypes. GWA studies have identified common genetic loci of small effect associated with clinical phenotypes such as coronary artery disease.^5^ The GWA method allows an agnostic study of variation within the genome, unbiased by prior knowledge of the cellular pathways inREFVIDved or the use of candidate genes, and has been successful in finding hundreds of gene loci to date.^6^ The overall aim was to determine whether genetic variants associated with coagulation and fibrin structure function were risk factors for ischemic stroke and if so whether such associations differed between stroke subtypes.

## Subjects and Methods

**FIGURE 1:**
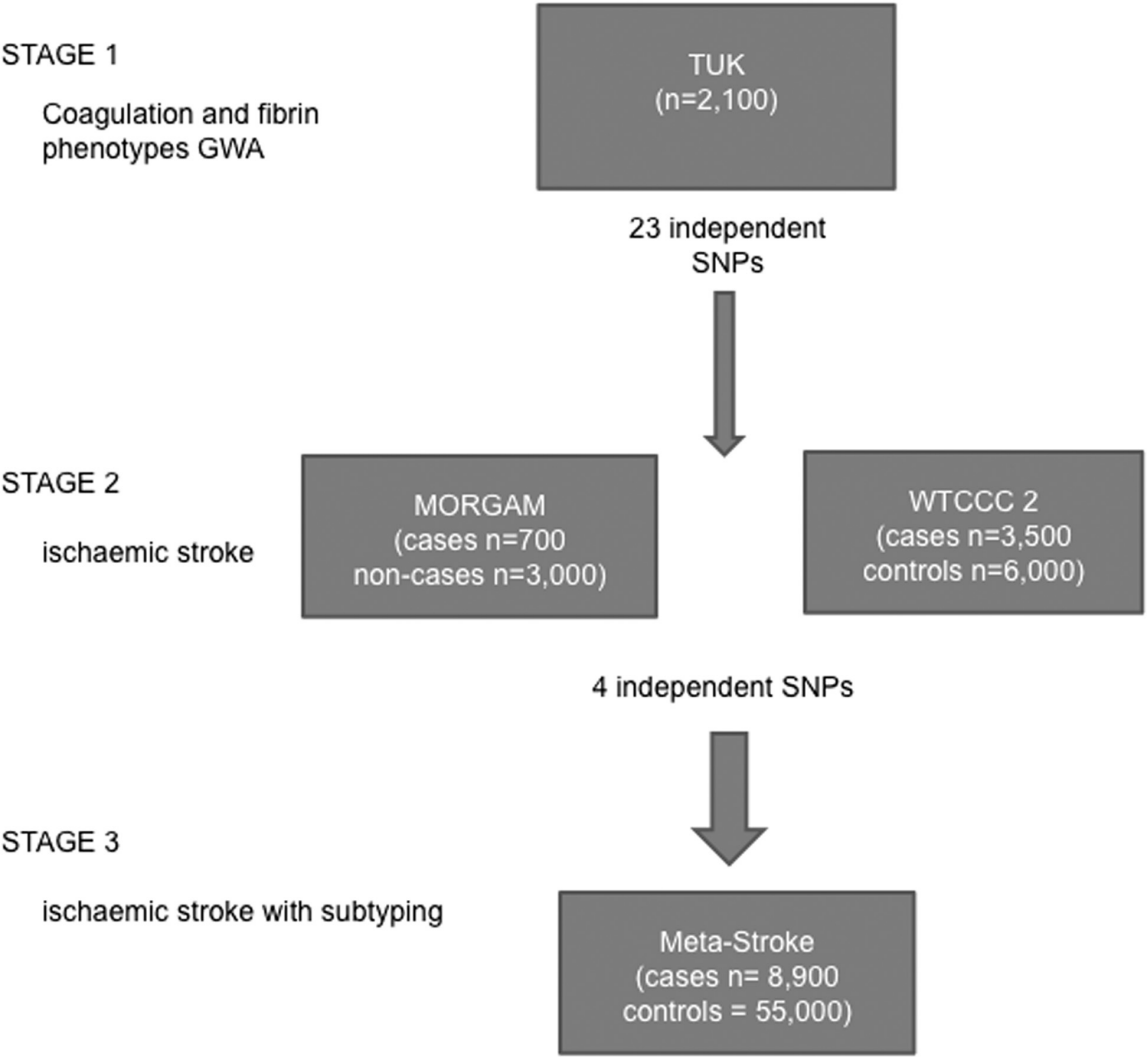
Flow chart showing study design and cohorts involved. The 3 stages of the study design are shown, with number of cases in each sample. GWA = genome-wide association; n = size of the cohort; SNP = single nucleotide polymorphism; TUK = TwinsUK; WTCCC2 = Wellcome Trust Case Control Consortium 2.

We used a 3-stage study design to identify common variants influencing coagulation and fibrin structure/function in the normal population and then tested genome-wide significant independent single nucleotide polymorphisms (SNPs) for association with stroke in subjects of Northern European extraction (Fig 1). To study the broad range of hemostatic variables contributing to end-stage coagulation, GWA studies of fibrin structure/function ex vivo, fibrin turnover (D-dimer) in vivo, and individual hemostatic components were performed in a healthy REFVIDunteer cohort of twins (Stage 1). In Stage 2, those variants found to be independently associated with coagulation or fibrin structure/function were assessed as risk factors for ischemic stroke in cases and controls. In Stage 3, the top 4 SNPs from the meta-analysis of ischemic stroke were examined for replication in a third clinical collection of stroke having information on whether stroke resulted from occlusion of large-vessel, small-vessel, or cardiac emboli. Detailed methods are provided below. Written informed consent was obtained from participants in the study, and each individual study group obtained local ethics approval.

## Phenotyping the Cohorts

TwinsUK. The subjects were obtained from the TwinsUK (TUK) registry (http://www.twinsuk.ac.uk) at King's College London, United Kingdom, which has been ascertained by a national media campaign.^7^ For historical reasons, the majority of twin REFVIDunteers are female. TUK subjects have been shown to be representative of the wider general populations for genetic and lifestyle factors associated with a variety of traits.^8^ TUK subjects were phenotyped for fibrin structure/function, D-dimer, and hemostatic factors, according to methods described in detail elsewhere.^9^–^12^ In brief, fibrin structure/function was assessed using a turbidimetric assay, whereas D-dimer (as a measure of in vivo fibrin turnover), coagulation factors (F) VII, VIII, FXII, FXIII A and B subunits (FXIIIA, FXIIIIB), prothrombin, and von Willebrand Factor (vWF) were quantified by enzyme-linked immunosorbent assay, and fibrinogen, FVII, and FXIII by functional activity assays.

The MOnica Risk, Genetics, Archiving and Monograph (MORGAM) Cohort. The cohorts of the MORGAM project consist of the respondents of representative adult population samples.^13^ This study includes cohorts from a variety of centers, including Finland (FINRISK, ATBC), France (Lille, Strasbourg, Toulouse), Italy (Brianza), Northern Sweden, and Northern Ireland (Belfast) as described at http://www.ktl.fi/publications/morgam/cohorts. The participants were examined and DNA was collected at baseline, and they were followed up for stroke and acute coronary events. Genotyping was carried out in a case–cohort setting.^14^ In MORGAM cohorts, the end-point used was the subject presenting with first ischemic stroke. For some events the diagnosis was based on validation, and for some on the clinical or death certificate diagnosis (International Classification of Diseases [ICD]-9 codes 433 or 434, or ICD-10 code I63).

Wellcome Trust Case Control Consortium 2. The Wellcome Trust Case Control Consortium 2 (WTCCC2) ischemic stroke study comprises ischemic stroke cases recruited from 3 centers in the United Kingdom (St George's London, Oxford, and Edinburgh) and 1 center in Munich, Germany. In all cases, ischemic stroke was defined as a focal neurological deficit lasting >24 hours; in 1 cohort (St George's), cases of transient ischemic attack with associated recent brain infraction were also included. Cerebral infraction was confirmed on brain imaging with computed tomography (CT) or magnetic resonance (MR) imaging, which was performed in 100% of cases, and extensive phenotyping was performed to allow stroke subtyping using a modified TOAST classification.^15^ Full details of populations and investigation performed have been previously published.^16^ Imaging of the cerebral arteries using carotid and vertebral duplex ultrasound and/or MR angiography or CT angiography was performed in >95%, echocardiography in 59.7%. Controls for the UK cases were the shared WTCCC2 controls drawn from the National Blood Service or the 1958 Birth Cohort Study (http://www.b58cgene.sgul.ac.uk). German controls werefrom the population-based KORAgen study (http://www.helmholtz-muenchen.de/en/kora-en/kora-homepage/index.html). This study group was used primarily in Stage 2 but also for subgroup analysis in Stage 3.

MetaStroke. MetaStroke is a project of the International Stroke Genetics Consortium and comprises ischemic stroke cases whose DNA has been collected and undergone GWA scan, recruited from centers in Europe (BRAINS [Bio-Repository of DNA in Stroke], United Kingdom; DeCODE, Iceland; Cerebrovascular Diseases Registry (CEDIR), Milan, Italy; Rotterdam, the Netherlands), USA (Atherosclerosis Risk in Communities study; Cardiovascular Health Study; Framingham Heart Study; Genetics of Early Onset Stroke Study; Heart Protection Study; Heart and Vascular Health; Ischemic Stroke Genetics Study; Massachusetts General Hospital Genes Affecting Stroke Risk and Outcome study), and Australia (Australian Stroke Genetics Collaborative). Ischemic stroke was defined clinically as a focal neurological deficit lasting >24 hours. In almost all case–control studies, a high level of brain imaging and extensive phenotyping was performed, although this was less detailed in some of the prospective studies. In those studies with adequate investigations to allow stroke subtyping, this was performed using a modified TOAST classification.^15^ Controls were collected by the individual groups.

## Genotyping and Within-Cohort Analysis

TUK. Genotyping was performed in 3 different genotypic batches using Human Hap 300 k Duo and Human Hap610 Quad array (Illumina, San Diego, CA). Genotyping results from the different arrays were collated and quality control was performed as described previously,^17^ including retention of those SNPs with sufficiently high genotyping rates (95% or above) and Hardy–Weinberg equilibrium (*p* > 0.0001). Imputation of nongenotyped SNPs was performed to HapMap2 Caucasian population haplotypes using IMPUTE version 2.^18^ Population substructure and admixture was excluded in TUK using Eigenvector analysis.

MORGAM. Four SNPS (rs10665, rs2022309, rs5985, and rs651007) were genotyped at the National Institute for Health and Welfare in Finland. Several sample- and plate-specific quality control measures were implemented to minimize errors, and in addition genotyping quality was assessed from 5% blind duplicate samples in each 96-well plate. For 234 samples with low DNA yield, DNA was amplified and genotyped as previously described.^19^ Genotyping was performed using the MassARRAY System and iPLEX Gold chemistry (Sequenom, San Diego, CA) with standard protocol. Genotype clusters were manually reviewed using Typer 4.0 software (Sequenom), and genotype calls were corrected where necessary. Genotyping success rate was >95% for all but 1 SNP (rs2022309, 91.3%), with an average success rate of 95.7%. No discrepancies were identified among a total of 1,256 successful blind duplicate genotype pairs. Cox regression analysis adapted for the case–cohort data was used to assess the association between the genotypes and ischemic stroke in the MORGAM cohorts, assuming an additive genotypic effect. The analysis was stratified by cohort and sex.

WTCCC2. Stroke cases were genotyped using the Illumina 660Q platform. Shared WTCCC2 controls were genotyped using the Illumina 1M Duo platform. German controls were genotyped using the Illumina 550 platform. Analysis of the UK and German cohorts was performed independently using PLINK^20^ after quality control checking using a genotyping call rate of 98%, Hardy–Weinberg equilibrium call rate of 1e ^–20^, and checks for individual relatedness and population stratification. The UK and German cases were then meta-analyzed using METAL.^21^ Samples were identified and removed if the genome-wide patterns of diversity differed from those of the collection at large, interpreting them as likely to be due to biases or artifacts. To do so, we used a Bayesian clustering approach to infer outlying individuals on the basis of call rate, heterozygosity, ancestry, and average probe intensity. We used a hidden Markov model to infer identity by descent along the genome and removed individuals iteratively to obtain a set with pair-wise identity by descent <5%. Samples were also removed if their inferred gender was discordant with the recorded gender or if <90% of the SNPs typed by Sequenom (Sequenom iPLEX assay for 4 gender SNPs) were concordant with the genome-wide data. For the EuroCLOT study, individual UK and German cohort and meta-analysis results were examined for the 23 available genotypes. This was performed for the phenotype of all ischemic stroke, together with the ischemic stroke subtypes of small-vessel disease, large-vessel disease, and cardioembolic stroke.

MetaStroke. Genotyping of the 13 MetaStroke contributors was performed independently by each group, using either Illumina or Affymetrix (Santa Clara, CA) platforms. Further details on cases and controls, genotyping, and imputation are available in Supplementary Table 3.

## Statistical Analysis

Stage 1. We used multiple linear regression models to assess association between genotypes and phenotypes, using age as a covariate. The phenotypes examined in the TUK cohort were inverse-normal transformed to satisfy the assumption of normality of trait distribution of the linear models. Association analysis was carried out using Merlin^22^ to control for family structure within the dataset. Independence of the effects conferred by SNPs in the same region was assessed by means of a backward stepwise regression analysis on the trait with which they were associated. This yielded 23 statistically independent significant SNPs (*p* < 5 × 10^–8^), associated with at least 1 quantitative outcome, which were taken forward for examination in the clinical groups at Stage 2. This stage of the analysis was performed using Stata for Windows version 10 (StataCorp, College Station, TX) with adjustment for the twins' relatedness.

Stage 2. The 23 independent SNPs remaining significant after multiple regression were carried through to investigation of association with ischemic stroke in MORGAM and WTCCC2. Results for each were meta-analyzed using a fixed effects inverse variance weighting implemented in METAL.^21^

Stage 3. The 4 most significantly associated SNPs from Stage 2 were tested for association with overall ischemic stroke in Meta-Stroke. This international collaboration brings together GWA studies in ischemic stroke and (depending on SNP) includes 8,900 cases of ischemic stroke and 55,000 controls. In addition, subgroup analysis was possible (in MetaStroke and WTCCC2), as stroke events had been subphenotyped into large-vessel, small-vessel, and cardioembolic stroke by many of the contributing study groups, using the TOAST classification.^15^ Within MetaStroke, samples were excluded from analysis if they had call rates <80% or if reported gender was discordant with gender-specific markers. We removed pairs of samples showing concordance indicative of being duplicates. MetaStroke genotyping results were imputed to HapMap2 using MACH2. Where SNPs were imputed, *r*^2^ values were >0.9. Four SNPs analyzed in these cohorts were meta-analyzed using a fixed effects model with the metan module in Stata version 10.

## Results

The characteristics of the 2,128 twin participants are shown for TUK in Table 1 and Supplementary Table 1 (Stage 1). The mean age of the twins was 50.4 years, and the sample included 87 (4.4%) males. All were of North European descent. The sample size varied between assays; for clarity, the number of subjects is included in the tables for each phenotype.

**TABLE 1:** Stage 1: Characteristics of the TwinsUK Discovery Sample

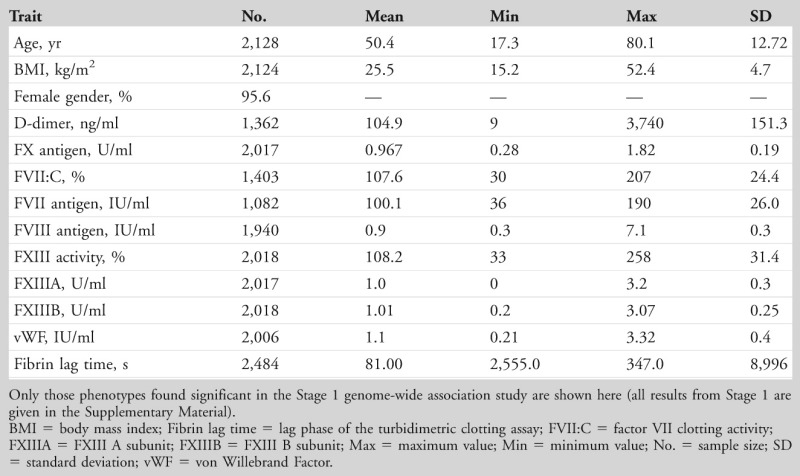

Details of the clinical collections of stroke cases and controls are shown in Table 2.

**TABLE 2:** Stages 2 and 3: Characteristics of the Ischemic Stroke Collections

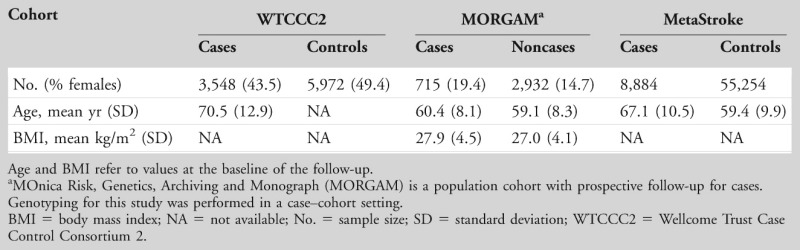

### Stage 1

There were a number of strikingly strong genotype–phenotype associations identified in the TUK discovery group, and in total 524 associations were found having *p* < 5 × 10^–8^. The 524 SNPs identified as significant genome-wide were mostly associated with coagulation factor phenotypes; there was 1 association with lag time to fibrin clot formation. After the interdependence of the SNPs had been established by backward stepwise regression analysis, 23 statistically independent SNPs were identified for examination in Stage 2 (shown in Table 3). The strongest signals were observed for SNP rs5985 in the *F13A1* gene (encoding the FXIII A subunit) and FXIII activity (*p* = 2.6 × 10^–186^), followed by rs2731672 in the *F12* gene associated with FXII concentration (encoding FXII; *p* = 1.3 × 10^–115^; Supplementary Table 2) and rs505922 in the *ABO* gene with vWF (*p* = 4.7 × 10^–57^; see Table 3) and factor VIII (*p* = 1.2 × 10^–36^; see Supplementary Table 2). Further coagulation-related phenotype–SNP associations were identified for rs10665 in *F7/MCF2L* and FVII clotting activity (*p* = 2.4 × 10^–47^), and rs2022309 in the *F3* gene (encoding tissue factor) with D-dimer concentration (*p* = 4.3 × 10^–8^).

**TABLE 3:** Stage 1: Independent SNPs (n = 23) Found with p < 5 × 10^-8^ in TwinsUK

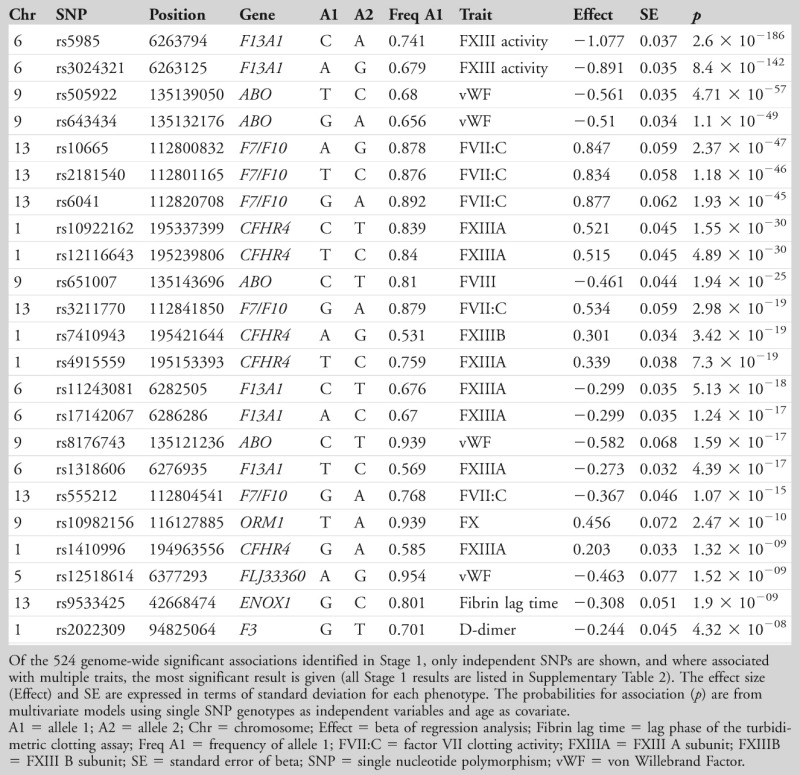

A clear relationship was found between plasma FXIII A subunit and SNP rs12137359 (*p* = 1.0 × 10^–27^) lying within the gene *ZBTB41* (zinc finger and BTB domain containing 41, a highly conserved gene). However, this region on chromosome 1q is rich with candidate genes, and the SNP in question lies downstream of the *CFH* and *CFHR1-5* genes (encoding complement factor H and CFH-related proteins 1 to 5) as well as *F13B* (encoding FXIII B subunit). There is also an association in this same region between rs800292 in the *CFH* gene and FXIIIA concentration (*p* = 1.5 × 10^–12^).

### Stage 2

In the MORGAM study, 6 of the 23 independent SNPs were available for lookup. None of the SNPs was significantly associated with ischemic stroke in this study group or in WTCCC2, although there was a suggestion of an effect for rs505922 in both MORGAM (T allele, beta = –0.126, *p* = 0.067) and WTCCC2 (T allele, beta = –0.054, *p* = 0.097). In the meta-analysis of WTCCC2 and MORGAM, SNP rs505922 in the *ABO* gene was associated with ischemic stroke (beta for T allele = –0.067, *p* = 0.023), with the major T allele being protective against stroke (Table 4).

**TABLE 4:** Stage 2: Meta-Analysis of the Independent SNPs in Stroke

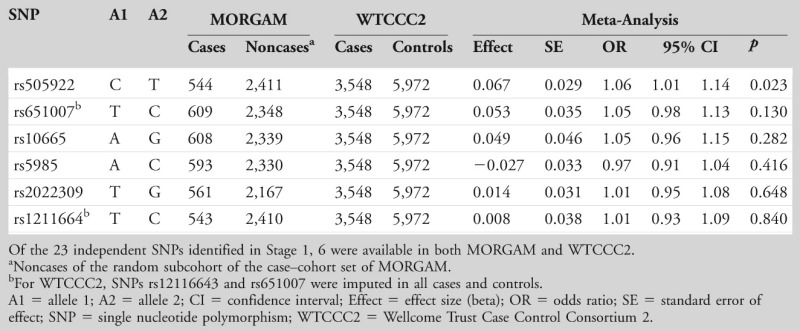

### Stage 3

**FIGURE 2:**
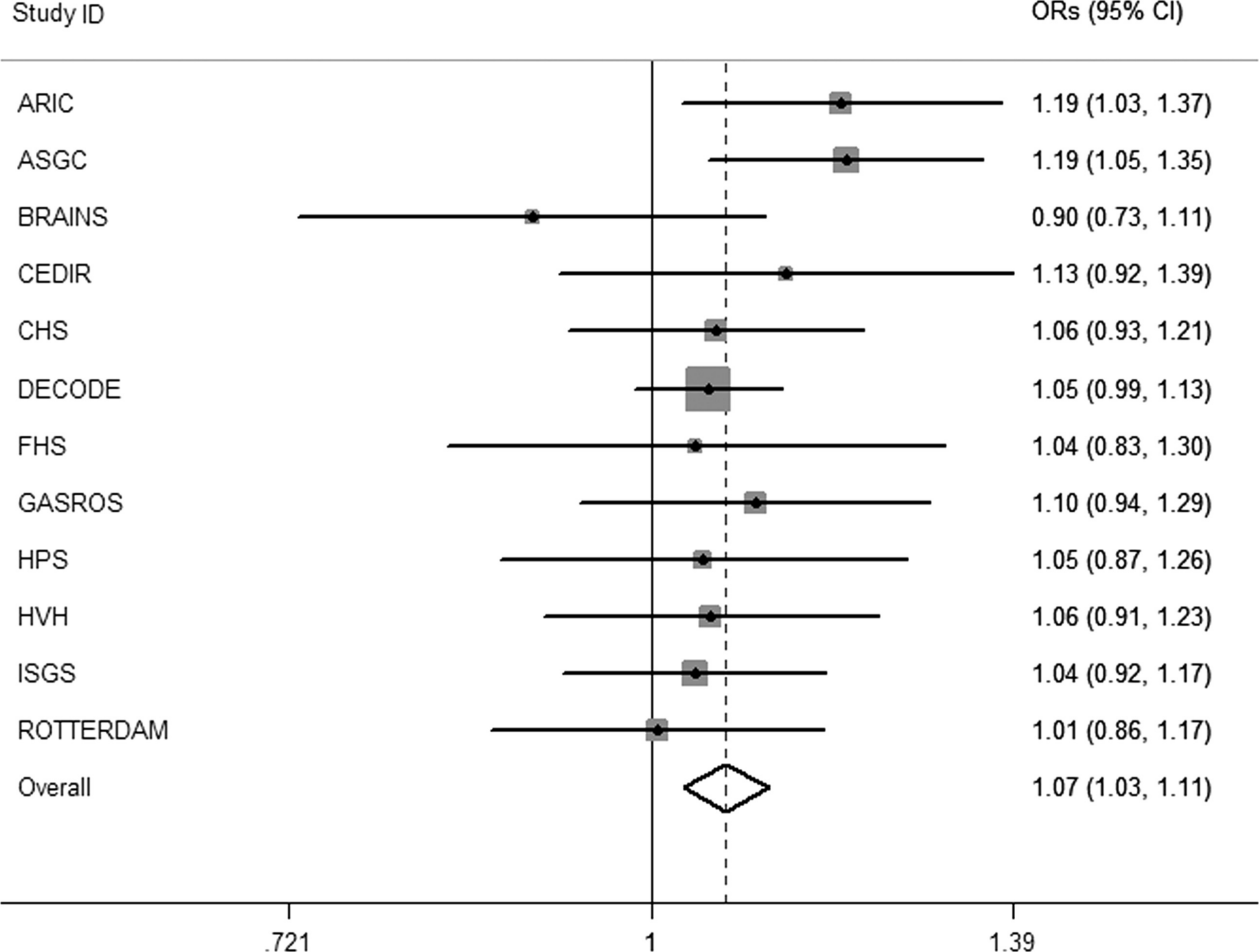
Forest plot shows results of the meta-analysis of rs505922 in Meta-Stroke. An inverse variance fixed effects model was used. The central filled dots represent odds ratios (ORs) in the individual cohorts, with their 95% confidence intervals (CIs; dark lines), and gray squares are proportional to sample size. The hollow diamond represents the meta-effect observed overall (p = 0.0006). ARIC = Atherosclerosis Risk in Communities; ASGC = Australian Stroke Genetics Collaborative; BRAINS = Bio- Repository of DNA in Stroke; CHS = Cardiovascular Health Study; FHS = Framingham Heart Study; GASROS = Genes Affecting Stroke Risk and Outcome Study; HPS = Heart Protection Study; HVH = Heart and Vascular Health; ISGS = Ischemic Stroke Genetics Study.

We examined the association of the 4 *ABO* SNPs in the large Meta-Stroke dataset (Table 5, with genotyping details in Supplementary Table 3) and further explored their relationship with stroke subtype in Meta-Stroke and WTCCC2. Results for ischemic stroke overall are shown in Table 6 (positive results) and are illustrated by a forest plot (Fig 2). The results show an association for lead SNP rs505922 C allele with ischemic stroke (odds ratio [OR] = 1.07, 95% confidence interval [CI] = 1.03–1.11, *p* = 0.0006). Two other *ABO* SNPs also showed significant association: rs643434 (for A allele, meta-analysis logistic regression OR = 1.06, 95% CI = 1.02–1.11, *p* = 0.002) and rs651007 (C allele, OR = 1.07, 95% CI = 1.02–1.12, *p* = 0.007; see Table 6). Analysis by stroke subtype for SNP rs505922 showed association with cardioembolic stroke (OR = 1.13, 95% CI = 1.11–1.15, *p* ≤ 0.001), and large-vessel stroke (OR = 1.23, 95% CI = 1.07–1.18, *p* = 0.001), but there was no association with small-vessel disease (*p* = 0.811; Table7).

**TABLE 5:** Characteristics of the Stroke Collections in the MetaStroke Consortium (Stage 3)

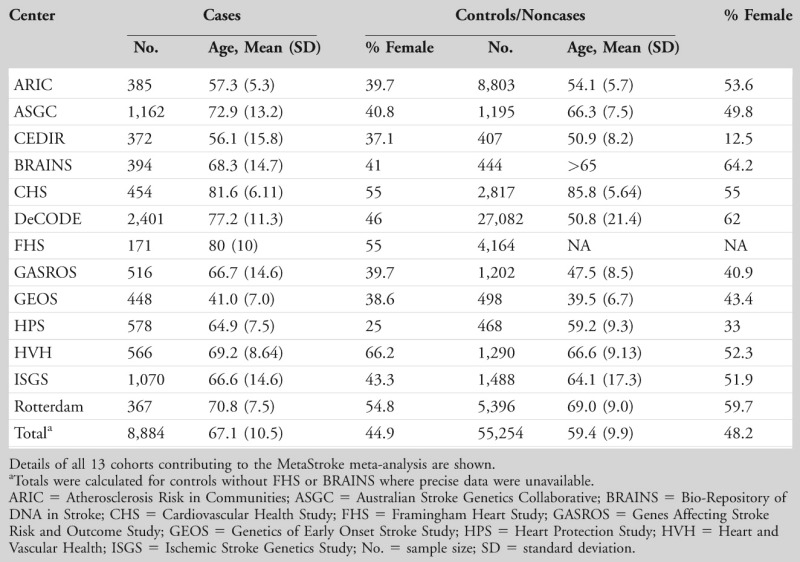

**TABLE 6:** Meta-Analysis of the ABO Locus in the MetaStroke Consortium: Stage 3

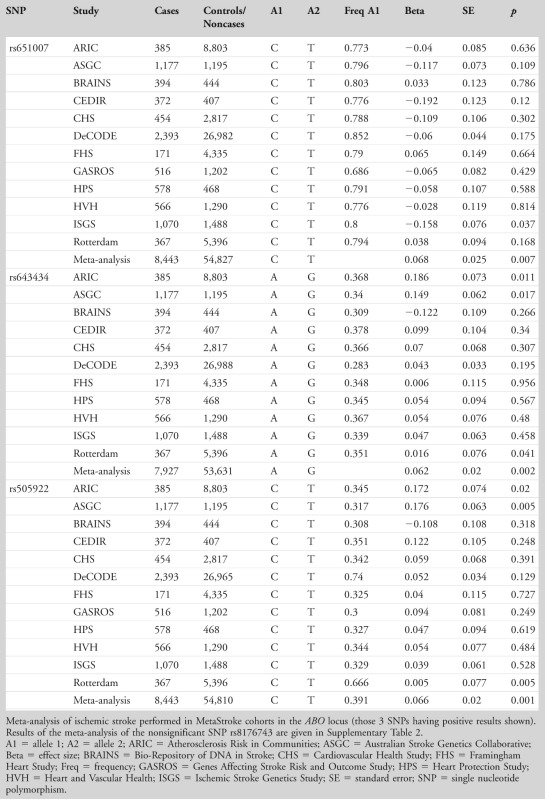

**TABLE 7:** Meta-Analysis of Single Nucleotide Polymorphism rs505922 in the *ABO* Locus by Stroke Subtype (Stage 3)

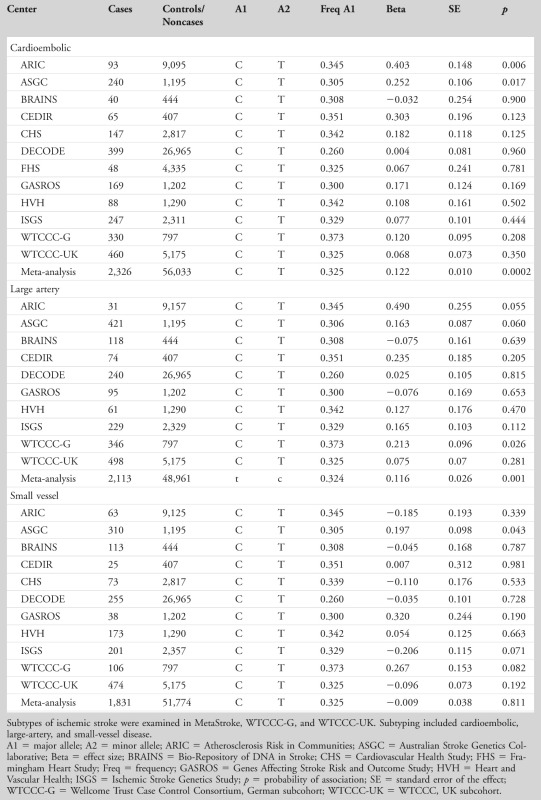

Finally, to determine whether the genetic influence was acting through known risk factors, we performed subgroup analysis in the sample having this information, WTCCC2–Munich. Adjusting for hypertension, hypercholesterolemia, diabetes, and smoking had a small effect on the strength of the association (unadjusted: beta = 0.159, 95% CI = 0.023–0.294, *p* = 0.022; adjusted: beta = 0.160, 95% CI = 0.010–0.309, *p* = 0.036).

## Discussion

Ischemic stroke accounts for considerable morbidity and mortality in Western countries, and treatment is limited at present. Our 3-stage study design optimized power for discovery of common genetic variants predisposing to ischemic stroke and stroke subtype. We performed a GWA study of intermediate coagulation and fibrinolytic phenotypes in healthy REFVIDunteers to examine the genetic determinants of end-stage coagulation and went on to study their influence on stroke and stroke subtype. We identified a large number of genetic variants associated with measures of coagulation factors, both functional and antigenic, some of which have been included in GWA meta-analyses of coagulation.^23^ We confirmed that polymorphisms in the *ABO* gene were significantly associated with vWF and FVIII levels in healthy REFVIDunteers. Significant associations between SNPs in *ABO* and levels of vWF (rs505922, rs643434, rs8176743) and/or FVIII (rs505922, rs651007) were identified; we went on to demonstrate significant associations between *ABO* SNPs, in particular rs505922, and ischemic stroke (see forest plot in Fig 2).

The associations between FVIII levels and the *ABO* gene variant rs505922, and between *ABO* and coronary disease, suggest a possible mechanism behind the well-documented association between the ABO blood group and risk of vascular disorders. Non-O blood groups are at increased risk of stroke,^24^ peripheral vascular disease, and myocardial infarction (MI) but not coronary artery disease (as assessed by angina, summarized by Wu et al^25^), and this suggests that end-stage coagulation is the critical determinant. The association we found with FVIII levels may account for this. Recent GWA studies of MI have identified variants within the *ABO* gene that predispose to MI,^26^, ^27^ and this relationship appears to hold for common forms of thrombotic stroke; we found evidence of association in large-vessel and cardioembolic stroke, but there was no association with small-vessel disease. At present, none of the SNPs significantly associated with stroke is reported to be associated with known risk factors such as hypertension, hyperlipidemia, diabetes, or propensity to drink alcohol or smoke. Subgroup analysis of the study group having risk factor information (WTCCC2–Munich) attenuated the strength of the association but did not suggest that the action of the genetic variation was predominantly though 1 of these risk factors.

SNP rs505922 represents a single base pair change from T to C at position 135,139,050 and lies within the first intron of the *ABO* gene, although its haplotype block contains the promoter and introns 1 and 2. The minor allele frequency of this SNP is 36% in Northern Europeans. The *ABO* gene encodes a glycosyltransferase enzyme that catalyses the transfer of different carbohydrate groups onto the H antigen, thus forming A and B antigens of the ABO system. In support of a functional role in thrombosis (as opposed to atherosclerosis), the non-O blood group has also been shown to be a risk factor for venous thrombosis,^28^ and in a large prospective study, pulmonary embolism.^29^ A previous GWA study identified the same SNP, rs505922, to be associated with venous thromboembolism,^30^ and a recent GWA study of blood metabolites suggests that this locus may act via an effect on fibrinogen phosphorylation.^31^

Our results demonstrate that the association between *ABO* SNPs and ischemic stroke is limited to large-artery and cardioembolic stroke, but absent in small-vessel stroke. Thromboembolism plays an important role in pathogenesis of both cardioembolic and large-artery stroke, with thrombus arising in the heart and on larger-artery atherosclerotic plaques, respectively, which may break off and embolize into the cerebral circulation. In both stroke subtypes, cerebral emboli can be detected in the cerebral circulation using transcranial Doppler,^32^ and antithromboembolic therapy reduces stroke risk. Recently, vWF inhibition has been shown to reduce cerebral thromboembolism in man,^33^ a clinical observation that is in keeping with our findings. In contrast, the pathogenesis of small-artery stroke is unclear, and the role of thrombosis remains uncertain.^34^ Our results suggest that thrombosis may be less important for this stroke subtype and explain why antithromboembolic medication is less effective. The subtype specificity we have identified is consistent with others' results; of 5 GWA studies identified and replicated, 2 have been studies of cardioembolic stroke,^35^,^36^ 2 of large-vessel stroke,^37^ and 1 of small-vessel stroke.^38^ Taken together, these data highlight that the clinical endpoint of ischemic stroke represents a varied phenotype likely resulting from multiple pathogenic mechanisms.

Other associations between SNPs and intermediate phenotypes included rs12137359 and FXIII activity and rs800292 and FXIIIA subunit levels. Both variants are found close to the gene encoding the FXIIIB subunit, which acts as a carrier protein for FXIIIA in the circulation and stabilizes FXIIIA to regulate activation; however, these SNPs were not associated with MI or ischemic stroke. We also identified associations between SNPs in the vicinity of the *F7* gene and FVII:C, consistent with a number of studies that have previously identified relationships between variation in the structural genes for FVII and circulating levels.^39^, ^40^ No other SNPs significantly associated with coagulation intermediate phenotypes were significantly associated with ischemic stroke.

There are a number of limitations to this work. First, TUK is predominantly female in its composition, for historical reasons. Although TUK subjects are representative of the general population variation^8^ and there is no evidence of an effect of gender on the ABO predisposition to cardiovascular disease, the associations identified in Stage 1 are pertinent to females from Northern Europe. Second, the clinical studies used for Stage 2 were heterogeneous in many respects. We decided that it was of overriding importance to obtain a large sample, so we combined prospective and cross-sectional studies. One of the main strengths of the study design was the use of multiple novel intermediate phenotypes, as well as having the power to investigate stroke subtypes. The Stage 3 study groups had differing methods of genotyping and imputation, but methods have been shown to be broadly comparable.^41^

In conclusion, using end-stage coagulation intermediate traits in healthy REFVIDunteers, we identified 23 genome-wide independent coagulation-associated SNPs, which were investigated in a number of clinical collections of stroke. Genetic variant rs505922 in the ABO locus was found to be associated with ischemic stroke, and in particular the subtypes large-vessel and cardioembolic stroke, but not small-vessel disease. This SNP was highly associated with vWF and FVIII in the discovery phase, and this observation throws light on possible mechanisms underlying end-stage coagulation in cardiovascular disease. It seems that common genetic variants exert some of their influence on end-stage stroke through coagulation, and further work is needed to tease apart these complex networks of interactions. The identification of the ABO locus through its association with vWF and FVIII points the way for mechanistic work to understand better the role of these 2 coagulation factors in end-stage arterial thrombosis.

## Appendix

### Membership of Wellcome Trust Case Control Consortium 2

#### Management Committee

Peter Donnelly (Chair), Wellcome Trust Centre for Human Genetics and Department of Statistics, University of Oxford, Oxford, United Kingdom; Ines Barroso (Deputy Chair), Wellcome Trust Genome Campus, Cambridge, United Kingdom; Jenefer M. Blackwell, Telethon Institute for Child Health Research, Child Health Research, University of Western Australia, Subiaco, Australia and Cambridge Institute for Medical Research, University of Cambridge School of Clinical Medicine, Cambridge, United Kingdom; Elvira Bramon, Department of Psychosis Studies, NIHR Biomedical Research Centre for Mental Health at the Institute of Psychiatry, King's College London and the South London and Maudsley NHS Foundation Trust, London, United Kingdom; Matthew A. Brown, University of Queensland Diamantina Institute, Brisbane, Australia; Juan P. Casas, Department Epidemiology and Population Health, London School of Hygiene and Tropical Medicine, Department of Epidemiology and Public Health, University College London, London, United Kingdom; Aiden Corvin, Neuropsychiatric Genetics Research Group, Institute of Molecular Medicine, Trinity College Dublin, Dublin, Ireland; Panos Deloukas, Wellcome Trust Genome Campus, Cambridge, United Kingdom; Audrey Duncanson, Molecular and Physiological Sciences, Wellcome Trust, London, United Kingdom; Janusz Jankowski, Department of Oncology, University of Oxford; Digestive Diseases Centre, Leicester Royal Infirmary, Leicester; and Centre for Digestive Diseases, Queen Mary University of London, London, United Kingdom; Hugh S. Markus, Stroke and Dementia Research Centre, St George's University of London, London, United Kingdom; Christopher G. Mathew, Department of Medical and Molecular Genetics, King's College London, King's Health Partners, and Guy's Hospital, London, United Kingdom; Colin N. A. Palmer, Biomedical Research Centre, Ninewells Hospital and Medical School, Dundee, United Kingdom; Robert Plomin, King's College London Social, Genetic, and Developmental Psychiatry Centre, Institute of Psychiatry, London, United Kingdom; Anna Rautanen, Wellcome Trust Centre for Human Genetics, University of Oxford, Oxford, United Kingdom; Stephen J. Sawcer, University of Cambridge Dept Clinical Neurosciences, Addenbrooke's Hospital, Cambridge, United Kingdom; Richard C. Trembath, Department of Medical and Molecular Genetics, King's College London, King's Health Partners, and Guy's Hospital, London, United Kingdom; Ananth C. Viswanathan, NIHR Biomedical Research Centre for Ophthalmology, Moorfields Eye Hospital NHS Foundation Trust, and UCL Institute of Ophthalmology, London, United Kingdom; and Nicholas W. Wood, Department of Molecular Neuroscience, Institute of Neurology, London, United Kingdom.

#### Data and Analysis Group

Chris C. A. Spencer, Gavin Band, Céline Bellenguez, Colin Freeman, Garrett Hellenthal, Eleni Giannoulatou, Matti Pirinen, Richard Pearson, Amy Strange, Zhan Su, and Damjan Vukcevic, Wellcome Trust Centre for Human Genetics, University of Oxford, Oxford, United Kingdom; and Peter Donnelly, Wellcome Trust Centre for Human Genetics and Department of Statistics, University of Oxford, Oxford, United Kingdom.

#### DNA, Genotyping, Data Quality Control, and Informatics Group

Cordelia Langford, Sarah E. Hunt, Sarah Edkins, Rhian Gwilliam, Hannah Blackburn, Suzannah J. Bumpstead, Serge Dronov, Matthew Gillman, Emma Gray, Naomi Hammond, Alagurevathi Jayakumar, Owen T. McCann, Jennifer Liddle, Simon C. Potter, Radhi Ravindrarajah, Michelle Ricketts, Matthew Waller, Paul Weston, Sara Widaa, Pamela Whittaker, Ines Barroso, and Panos Deloukas, Wellcome Trust Genome Campus, Cambridge, United Kingdom.

#### Publications Committee

Christopher G. Mathew (Chair), Department of Medical and Molecular Genetics, King's College London, King's Health Partners, and Guy's Hospital, London, United Kingdom; Jenefer M. Blackwell, Telethon Institute for Child Health Research, Child Health Research, University of Western Australia, Subiaco, Australia and Cambridge Institute for Medical Research, University of Cambridge School of Clinical Medicine, Cambridge, United Kingdom; Matthew A. Brown, University of Queensland Diamantina Institute, Brisbane, Australia; Aiden Corvin, Neuropsychiatric Genetics Research Group, Institute of Molecular Medicine, Trinity College Dublin, Dublin, Ireland; and Chris C. A. Spencer, Wellcome Trust Centre for Human Genetics, University of Oxford, Oxford, United Kingdom.
