# Supplementary material for: Ischemic Stroke Is Associated with the ABO Locus: The EuroCLOT Study
Source: Ann Neurol. 2013 Feb 4;73(1):16–31. doi: 10.1002/ana.23838 (PMC3582024; doi:10.1002/ana.23838)
Supplement: Supplementary file 1 [file ana0073-0016-SD1.doc]

**Supplementary Table 1. Characteristics of coagulation and fibrin phenotypes having non-significant GWA results in Stage 1**

| **Trait** | N | Min | Max | Mean | SD |
| --- | --- | --- | --- | --- | --- |
| FXII (ng/ml) | 2014 | 0.03 | 24.6 | 2.5 | 1.16 |
| Fibrinogen (g/L) | 2021 | 0 | 9.65 | 3.0 | 0.82 |
| PAI (ng/ml) | 2012 | 0.4 | 177.2 | 11.49 | 13.34 |
| PLGAG (u/ml) | 2006 | 0.4 | 1.77 | 0.98 | 0.19 |
| Prothrombin (u/ml) | 2015 | 0.35 | 1.9 | 0.95 | 0.17 |
| tPA (ng/ml) | 1994 | 0 | 26.6 | 6.73 | 3.58 |

**Legend to Supplementary Table 1**

Those traits are shown having non-significant findings in Stage 1 GWA

FXII represents coagulation factor XII

PAI plasminogen activator inhibitor

tPA tissue plasminogen activator

N represents sample size, min minimum value, max maximum value, SD standard deviation

| CHR | SNP | AL1 | AL2 | FREQ | TRAIT | EFFECT | SE | p |
| --- | --- | --- | --- | --- | --- | --- | --- | --- |
| 1 | rs4915344 | A | G | 0.839 | FXIIIA | 0.521 | 0.05 | 1.537E-30 |
| 1 | rs12042442 | T | C | 0.839 | FXIIIA | 0.521 | 0.05 | 1.541E-30 |
| 1 | rs10922168 | T | C | 0.839 | FXIIIA | 0.521 | 0.05 | 1.545E-30 |
| 1 | rs12034362 | T | C | 0.839 | FXIIIA | 0.521 | 0.05 | 1.547E-30 |
| 1 | rs12085377 | G | A | 0.839 | FXIIIA | 0.521 | 0.05 | 1.547E-30 |
| 1 | rs10922162 | C | T | 0.839 | FXIIIA | 0.521 | 0.05 | 1.548E-30 |
| 1 | rs10737686 | C | T | 0.842 | FXIIIA | 0.523 | 0.05 | 1.584E-30 |
| 1 | rs2298883 | A | G | 0.843 | FXIIIA | 0.523 | 0.05 | 1.91E-30 |
| 1 | rs10801586 | C | T | 0.843 | FXIIIA | 0.523 | 0.05 | 1.955E-30 |
| 1 | rs12116643 | T | C | 0.84 | FXIIIA | 0.515 | 0.05 | 4.894E-30 |
| 1 | rs12075161 | T | A | 0.84 | FXIIIA | 0.513 | 0.05 | 5.901E-30 |
| 1 | rs12134960 | G | C | 0.84 | FXIIIA | 0.513 | 0.05 | 5.901E-30 |
| 1 | rs1332660 | T | C | 0.84 | FXIIIA | 0.52 | 0.05 | 6.066E-30 |
| 1 | rs4639796 | G | A | 0.839 | FXIIIA | 0.493 | 0.05 | 8.085E-28 |
| 1 | rs12028827 | A | C | 0.839 | FXIIIA | 0.493 | 0.05 | 8.089E-28 |
| 1 | rs12137359 | C | T | 0.839 | FXIIIA | 0.492 | 0.05 | 1.022E-27 |
| 1 | rs12128631 | G | A | 0.839 | FXIIIA | 0.491 | 0.05 | 1.323E-27 |
| 1 | rs12075161 | T | A | 0.84 | FXIIIB | 0.47 | 0.05 | 6.956E-26 |
| 1 | rs12134960 | G | C | 0.84 | FXIIIB | 0.47 | 0.05 | 6.956E-26 |
| 1 | rs12116643 | T | C | 0.84 | FXIIIB | 0.47 | 0.05 | 7.558E-26 |
| 1 | rs2298883 | A | G | 0.843 | FXIIIB | 0.471 | 0.05 | 1.515E-25 |
| 1 | rs10801586 | C | T | 0.843 | FXIIIB | 0.471 | 0.05 | 1.547E-25 |
| 1 | rs4915344 | A | G | 0.839 | FXIIIB | 0.464 | 0.05 | 4.746E-25 |
| 1 | rs12042442 | T | C | 0.839 | FXIIIB | 0.464 | 0.05 | 4.773E-25 |
| 1 | rs10922168 | T | C | 0.839 | FXIIIB | 0.464 | 0.05 | 4.797E-25 |
| 1 | rs12085377 | G | A | 0.839 | FXIIIB | 0.464 | 0.05 | 4.804E-25 |
| 1 | rs12034362 | T | C | 0.839 | FXIIIB | 0.464 | 0.05 | 4.809E-25 |
| 1 | rs10922162 | C | T | 0.839 | FXIIIB | 0.464 | 0.05 | 4.811E-25 |
| 1 | rs10737686 | C | T | 0.842 | FXIIIB | 0.464 | 0.05 | 6.236E-25 |
| 1 | rs1332660 | T | C | 0.84 | FXIIIB | 0.46 | 0.05 | 2.51E-24 |
| 1 | rs4639796 | G | A | 0.839 | FXIIIB | 0.445 | 0.05 | 2.129E-23 |
| 1 | rs12028827 | A | C | 0.839 | FXIIIB | 0.445 | 0.05 | 2.13E-23 |
| 1 | rs12128631 | G | A | 0.839 | FXIIIB | 0.442 | 0.05 | 3.69E-23 |
| 1 | rs12137359 | C | T | 0.839 | FXIIIB | 0.441 | 0.05 | 4.413E-23 |
| 1 | rs1412635 | G | A | 0.53 | FXIIIB | 0.318 | 0.03 | 7.724E-22 |
| 1 | rs7518773 | A | G | 0.525 | FXIIIB | 0.314 | 0.03 | 1.22E-21 |
| 1 | rs1332667 | G | T | 0.525 | FXIIIB | 0.314 | 0.03 | 1.29E-21 |
| 1 | rs10737685 | A | G | 0.525 | FXIIIB | 0.314 | 0.03 | 1.29E-21 |
| 1 | rs6702252 | G | A | 0.525 | FXIIIB | 0.314 | 0.03 | 1.29E-21 |
| 1 | rs1332668 | C | T | 0.525 | FXIIIB | 0.314 | 0.03 | 1.29E-21 |
| 1 | rs5990 | A | C | 0.525 | FXIIIB | 0.314 | 0.03 | 1.29E-21 |
| 1 | rs5998 | A | G | 0.525 | FXIIIB | 0.314 | 0.03 | 1.29E-21 |
| 1 | rs10484502 | G | A | 0.525 | FXIIIB | 0.314 | 0.03 | 1.29E-21 |
| 1 | rs10754209 | T | A | 0.525 | FXIIIB | 0.314 | 0.03 | 1.29E-21 |
| 1 | rs1611982 | G | A | 0.525 | FXIIIB | 0.314 | 0.03 | 1.29E-21 |
| 1 | rs1615413 | A | G | 0.525 | FXIIIB | 0.314 | 0.03 | 1.29E-21 |
| 1 | rs2298882 | T | G | 0.525 | FXIIIB | 0.314 | 0.03 | 1.29E-21 |
| 1 | rs10801589 | C | T | 0.524 | FXIIIB | 0.311 | 0.03 | 3.827E-21 |
| 1 | rs10922163 | T | C | 0.524 | FXIIIB | 0.311 | 0.03 | 4.149E-21 |
| 1 | rs6700180 | G | A | 0.518 | FXIIIB | 0.311 | 0.03 | 1.321E-20 |
| 1 | rs4244139 | G | A | 0.534 | FXIIIB | 0.304 | 0.03 | 9.621E-20 |
| 1 | rs6677930 | C | A | 0.528 | FXIIIB | 0.301 | 0.03 | 1.354E-19 |
| 1 | rs7410943 | A | G | 0.531 | FXIIIB | 0.301 | 0.03 | 3.422E-19 |
| 1 | rs10922147 | C | G | 0.759 | FXIIIA | 0.34 | 0.04 | 3.881E-19 |
| 1 | rs4915559 | T | C | 0.759 | FXIIIA | 0.339 | 0.04 | 7.302E-19 |
| 1 | rs7531555 | C | T | 0.767 | FXIIIA | 0.345 | 0.04 | 1.009E-18 |
| 1 | rs3790414 | T | A | 0.768 | FXIIIA | 0.345 | 0.04 | 1.174E-18 |
| 1 | rs4085749 | C | T | 0.768 | FXIIIA | 0.345 | 0.04 | 1.175E-18 |
| 1 | rs12066959 | G | A | 0.768 | FXIIIA | 0.345 | 0.04 | 1.187E-18 |
| 1 | rs10801582 | G | A | 0.769 | FXIIIA | 0.344 | 0.04 | 1.987E-18 |
| 1 | rs4915318 | C | A | 0.758 | FXIIIA | 0.332 | 0.04 | 2.531E-18 |
| 1 | rs4915559 | T | C | 0.759 | FXIIIB | 0.316 | 0.04 | 5.835E-17 |
| 1 | rs4915318 | C | A | 0.758 | FXIIIB | 0.312 | 0.04 | 1.109E-16 |
| 1 | rs7531555 | C | T | 0.767 | FXIIIB | 0.32 | 0.04 | 1.186E-16 |
| 1 | rs10922147 | C | G | 0.759 | FXIIIB | 0.311 | 0.04 | 1.353E-16 |
| 1 | rs4085749 | C | T | 0.768 | FXIIIB | 0.319 | 0.04 | 1.649E-16 |
| 1 | rs3790414 | T | A | 0.768 | FXIIIB | 0.319 | 0.04 | 1.649E-16 |
| 1 | rs12066959 | G | A | 0.768 | FXIIIB | 0.319 | 0.04 | 1.654E-16 |
| 1 | rs10801582 | G | A | 0.769 | FXIIIB | 0.316 | 0.04 | 3.597E-16 |
| 1 | rs7366238 | A | G | 0.768 | FXIIIA | 0.313 | 0.04 | 7.287E-16 |
| 1 | rs12116643 | T | C | 0.84 | FXIII activity | 0.36 | 0.05 | 2.647E-15 |
| 1 | rs2298883 | A | G | 0.843 | FXIII activity | 0.362 | 0.05 | 2.826E-15 |
| 1 | rs10801586 | C | T | 0.843 | FXIII activity | 0.362 | 0.05 | 2.901E-15 |
| 1 | rs7366238 | A | G | 0.768 | FXIIIB | 0.302 | 0.04 | 3.227E-15 |
| 1 | rs12075161 | T | A | 0.84 | FXIII activity | 0.356 | 0.05 | 4.091E-15 |
| 1 | rs12134960 | G | C | 0.84 | FXIII activity | 0.356 | 0.05 | 4.091E-15 |
| 1 | rs10737686 | C | T | 0.842 | FXIII activity | 0.355 | 0.05 | 8.436E-15 |
| 1 | rs1332660 | T | C | 0.84 | FXIII activity | 0.354 | 0.05 | 1.455E-14 |
| 1 | rs10922162 | C | T | 0.839 | FXIII activity | 0.344 | 0.05 | 4.244E-14 |
| 1 | rs12034362 | T | C | 0.839 | FXIII activity | 0.344 | 0.05 | 4.245E-14 |
| 1 | rs12085377 | G | A | 0.839 | FXIII activity | 0.344 | 0.05 | 4.249E-14 |
| 1 | rs10922168 | T | C | 0.839 | FXIII activity | 0.344 | 0.05 | 4.255E-14 |
| 1 | rs12042442 | T | C | 0.839 | FXIII activity | 0.344 | 0.05 | 4.274E-14 |
| 1 | rs4915344 | A | G | 0.839 | FXIII activity | 0.344 | 0.05 | 4.297E-14 |
| 1 | rs10922144 | C | T | 0.784 | FXIIIA | 0.292 | 0.04 | 1.205E-13 |
| 1 | rs12137359 | C | T | 0.839 | FXIII activity | 0.336 | 0.05 | 1.242E-13 |
| 1 | rs12128631 | G | A | 0.839 | FXIII activity | 0.335 | 0.05 | 1.406E-13 |
| 1 | rs10922146 | C | T | 0.78 | FXIIIA | 0.288 | 0.04 | 1.436E-13 |
| 1 | rs12047098 | T | C | 0.78 | FXIIIA | 0.288 | 0.04 | 1.436E-13 |
| 1 | rs7413265 | G | T | 0.78 | FXIIIA | 0.288 | 0.04 | 1.437E-13 |
| 1 | rs4639796 | G | A | 0.839 | FXIII activity | 0.335 | 0.05 | 1.543E-13 |
| 1 | rs12028827 | A | C | 0.839 | FXIII activity | 0.335 | 0.05 | 1.543E-13 |
| 1 | rs1963605 | A | C | 0.778 | FXIIIA | 0.288 | 0.04 | 1.747E-13 |
| 1 | rs7516246 | T | C | 0.684 | FXIIIB | -0.269 | 0.04 | 2.106E-13 |
| 1 | rs10801596 | A | G | 0.684 | FXIIIB | -0.269 | 0.04 | 2.132E-13 |
| 1 | rs2284664 | C | T | 0.785 | FXIIIA | 0.292 | 0.04 | 2.383E-13 |
| 1 | rs1964645 | C | T | 0.783 | FXIIIA | 0.287 | 0.04 | 2.761E-13 |
| 1 | rs10801560 | C | A | 0.785 | FXIIIA | 0.292 | 0.04 | 2.802E-13 |
| 1 | rs10801561 | T | A | 0.785 | FXIIIA | 0.292 | 0.04 | 2.802E-13 |
| 1 | rs6657442 | T | C | 0.779 | FXIIIA | 0.281 | 0.04 | 3.769E-13 |
| 1 | rs1360558 | G | A | 0.587 | FXIIIB | -0.258 | 0.04 | 4.274E-13 |
| 1 | rs10922146 | C | T | 0.78 | FXIIIB | 0.279 | 0.04 | 4.303E-13 |
| 1 | rs12047098 | T | C | 0.78 | FXIIIB | 0.279 | 0.04 | 4.303E-13 |
| 1 | rs7413265 | G | T | 0.78 | FXIIIB | 0.279 | 0.04 | 4.305E-13 |
| 1 | rs4657825 | G | A | 0.787 | FXIIIA | 0.297 | 0.04 | 5.286E-13 |
| 1 | rs529825 | G | A | 0.776 | FXIIIA | 0.284 | 0.04 | 6.913E-13 |
| 1 | rs1831281 | C | T | 0.784 | FXIIIA | 0.29 | 0.04 | 7.198E-13 |
| 1 | rs1963605 | A | C | 0.778 | FXIIIB | 0.275 | 0.04 | 9.102E-13 |
| 1 | rs3737110 | C | A | 0.589 | FXIIIB | -0.251 | 0.04 | 1.14E-12 |
| 1 | rs800292 | G | A | 0.768 | FXIIIA | 0.273 | 0.04 | 1.457E-12 |
| 1 | rs12061508 | G | A | 0.79 | FXIIIA | 0.292 | 0.04 | 1.464E-12 |
| 1 | rs10922144 | C | T | 0.784 | FXIIIB | 0.275 | 0.04 | 1.673E-12 |
| 1 | rs5996 | C | G | 0.574 | FXIIIB | -0.233 | 0.03 | 1.888E-12 |
| 1 | rs1964645 | C | T | 0.783 | FXIIIB | 0.273 | 0.04 | 1.978E-12 |
| 1 | rs698859 | C | T | 0.574 | FXIIIB | -0.232 | 0.03 | 2.271E-12 |
| 1 | rs857025 | C | T | 0.574 | FXIIIB | -0.232 | 0.03 | 2.271E-12 |
| 1 | rs6680396 | A | G | 0.779 | FXIIIA | 0.279 | 0.04 | 2.599E-12 |
| 1 | rs6657442 | T | C | 0.779 | FXIIIB | 0.266 | 0.04 | 3.124E-12 |
| 1 | rs7531555 | C | T | 0.767 | FXIII activity | 0.272 | 0.04 | 4.262E-12 |
| 1 | rs12124794 | A | T | 0.785 | FXIIIA | 0.281 | 0.04 | 4.378E-12 |
| 1 | rs4085749 | C | T | 0.768 | FXIII activity | 0.271 | 0.04 | 5.391E-12 |
| 1 | rs3790414 | T | A | 0.768 | FXIII activity | 0.271 | 0.04 | 5.391E-12 |
| 1 | rs12066959 | G | A | 0.768 | FXIII activity | 0.271 | 0.04 | 5.407E-12 |
| 1 | rs10801582 | G | A | 0.769 | FXIII activity | 0.272 | 0.04 | 5.655E-12 |
| 1 | rs10494747 | T | A | 0.724 | FXIIIB | -0.263 | 0.04 | 6.976E-12 |
| 1 | rs10754218 | A | G | 0.724 | FXIIIB | -0.262 | 0.04 | 7.794E-12 |
| 1 | rs4915318 | C | A | 0.758 | FXIII activity | 0.261 | 0.04 | 8.36E-12 |
| 1 | rs2284664 | C | T | 0.785 | FXIIIB | 0.269 | 0.04 | 9.279E-12 |
| 1 | rs3762271 | G | T | 0.577 | FXIIIB | -0.225 | 0.03 | 1.048E-11 |
| 1 | rs12029571 | A | G | 0.777 | FXIIIA | 0.267 | 0.04 | 1.524E-11 |
| 1 | rs12041668 | C | T | 0.779 | FXIIIA | 0.276 | 0.04 | 1.529E-11 |
| 1 | rs10801560 | C | A | 0.785 | FXIIIB | 0.266 | 0.04 | 1.565E-11 |
| 1 | rs10801561 | T | A | 0.785 | FXIIIB | 0.266 | 0.04 | 1.565E-11 |
| 1 | rs10922147 | C | G | 0.759 | FXIII activity | 0.257 | 0.04 | 1.698E-11 |
| 1 | rs4915559 | T | C | 0.759 | FXIII activity | 0.257 | 0.04 | 2.126E-11 |
| 1 | rs1412635 | G | A | 0.53 | FXIIIA | 0.224 | 0.03 | 2.45E-11 |
| 1 | rs10801551 | G | A | 0.782 | FXIIIA | 0.269 | 0.04 | 2.795E-11 |
| 1 | rs955927 | A | T | 0.579 | FXIIIB | -0.222 | 0.03 | 2.866E-11 |
| 1 | rs1332667 | G | T | 0.525 | FXIIIA | 0.219 | 0.03 | 4.953E-11 |
| 1 | rs10737685 | A | G | 0.525 | FXIIIA | 0.219 | 0.03 | 4.953E-11 |
| 1 | rs6702252 | G | A | 0.525 | FXIIIA | 0.219 | 0.03 | 4.953E-11 |
| 1 | rs1332668 | C | T | 0.525 | FXIIIA | 0.219 | 0.03 | 4.953E-11 |
| 1 | rs5990 | A | C | 0.525 | FXIIIA | 0.219 | 0.03 | 4.953E-11 |
| 1 | rs5998 | A | G | 0.525 | FXIIIA | 0.219 | 0.03 | 4.953E-11 |
| 1 | rs10484502 | G | A | 0.525 | FXIIIA | 0.219 | 0.03 | 4.953E-11 |
| 1 | rs10754209 | T | A | 0.525 | FXIIIA | 0.219 | 0.03 | 4.953E-11 |
| 1 | rs1611982 | G | A | 0.525 | FXIIIA | 0.219 | 0.03 | 4.953E-11 |
| 1 | rs1615413 | A | G | 0.525 | FXIIIA | 0.219 | 0.03 | 4.953E-11 |
| 1 | rs2298882 | T | G | 0.525 | FXIIIA | 0.219 | 0.03 | 4.953E-11 |
| 1 | rs1831281 | C | T | 0.784 | FXIIIB | 0.263 | 0.04 | 5.32E-11 |
| 1 | rs10922163 | T | C | 0.524 | FXIIIA | 0.219 | 0.03 | 6.045E-11 |
| 1 | rs10801589 | C | T | 0.524 | FXIIIA | 0.218 | 0.03 | 6.214E-11 |
| 1 | rs1127661 | T | C | 0.577 | FXIIIB | -0.217 | 0.03 | 6.413E-11 |
| 1 | rs7518773 | A | G | 0.525 | FXIIIA | 0.217 | 0.03 | 6.873E-11 |
| 1 | rs12124794 | A | T | 0.785 | FXIIIB | 0.261 | 0.04 | 7.084E-11 |
| 1 | rs4915406 | T | C | 0.68 | FXIIIB | -0.231 | 0.04 | 7.854E-11 |
| 1 | rs12061508 | G | A | 0.79 | FXIIIB | 0.265 | 0.04 | 8.256E-11 |
| 1 | rs7542212 | G | A | 0.68 | FXIIIB | -0.231 | 0.04 | 8.538E-11 |
| 1 | rs6690022 | G | A | 0.68 | FXIIIB | -0.23 | 0.04 | 1.145E-10 |
| 1 | rs7544067 | T | C | 0.68 | FXIIIB | -0.23 | 0.04 | 1.146E-10 |
| 1 | rs1415216 | T | C | 0.68 | FXIIIB | -0.23 | 0.04 | 1.147E-10 |
| 1 | rs3790380 | A | C | 0.68 | FXIIIB | -0.229 | 0.04 | 1.183E-10 |
| 1 | rs10922187 | T | C | 0.681 | FXIIIB | -0.228 | 0.04 | 1.595E-10 |
| 1 | rs4244139 | G | A | 0.534 | FXIIIA | 0.215 | 0.03 | 2.212E-10 |
| 1 | rs7366238 | A | G | 0.768 | FXIII activity | 0.246 | 0.04 | 2.827E-10 |
| 1 | rs12041668 | C | T | 0.779 | FXIIIB | 0.256 | 0.04 | 2.838E-10 |
| 1 | rs7410943 | A | G | 0.531 | FXIIIA | 0.215 | 0.03 | 2.848E-10 |
| 1 | rs12029571 | A | G | 0.777 | FXIIIB | 0.245 | 0.04 | 3.581E-10 |
| 1 | rs4657825 | G | A | 0.787 | FXIIIB | 0.254 | 0.04 | 4.139E-10 |
| 1 | rs12138336 | C | G | 0.957 | FXIIIA | 0.569 | 0.09 | 4.79E-10 |
| 1 | rs12755054 | T | C | 0.676 | FXIIIB | -0.218 | 0.04 | 6.05E-10 |
| 1 | rs1759016 | C | T | 0.676 | FXIIIB | -0.218 | 0.04 | 6.05E-10 |
| 1 | rs1750311 | C | A | 0.676 | FXIIIB | -0.218 | 0.04 | 6.05E-10 |
| 1 | rs7555070 | T | C | 0.677 | FXIIIB | -0.218 | 0.04 | 6.396E-10 |
| 1 | rs1329428 | C | T | 0.59 | FXIIIA | 0.204 | 0.03 | 6.889E-10 |
| 1 | rs6700180 | G | A | 0.518 | FXIIIA | 0.208 | 0.03 | 6.894E-10 |
| 1 | rs7540032 | C | T | 0.59 | FXIIIA | 0.202 | 0.03 | 9.02E-10 |
| 1 | rs6677930 | C | A | 0.528 | FXIIIA | 0.206 | 0.03 | 9.821E-10 |
| 1 | rs1410996 | G | A | 0.585 | FXIIIA | 0.203 | 0.03 | 1.322E-09 |
| 1 | rs10922106 | A | G | 0.585 | FXIIIA | 0.203 | 0.03 | 1.327E-09 |
| 1 | rs3753395 | A | T | 0.585 | FXIIIA | 0.203 | 0.03 | 1.328E-09 |
| 1 | rs10737680 | A | C | 0.585 | FXIIIA | 0.203 | 0.03 | 1.329E-09 |
| 1 | rs10801551 | G | A | 0.782 | FXIIIB | 0.242 | 0.04 | 1.361E-09 |
| 1 | rs6680396 | A | G | 0.779 | FXIIIB | 0.238 | 0.04 | 1.493E-09 |
| 1 | rs10922146 | C | T | 0.78 | FXIII activity | 0.235 | 0.04 | 1.985E-09 |
| 1 | rs12047098 | T | C | 0.78 | FXIII activity | 0.235 | 0.04 | 1.985E-09 |
| 1 | rs7413265 | G | T | 0.78 | FXIII activity | 0.235 | 0.04 | 1.985E-09 |
| 1 | rs529825 | G | A | 0.776 | FXIIIB | 0.233 | 0.04 | 2.404E-09 |
| 1 | rs12125782 | A | T | 0.959 | FXIIIA | 0.544 | 0.09 | 2.434E-09 |
| 1 | rs1963605 | A | C | 0.778 | FXIII activity | 0.234 | 0.04 | 2.535E-09 |
| 1 | rs1964645 | C | T | 0.783 | FXIII activity | 0.233 | 0.04 | 3.851E-09 |
| 1 | rs10801560 | C | A | 0.785 | FXIII activity | 0.236 | 0.04 | 3.913E-09 |
| 1 | rs10801561 | T | A | 0.785 | FXIII activity | 0.236 | 0.04 | 3.913E-09 |
| 1 | rs10922177 | T | C | 0.838 | FXIIIA | 0.556 | 0.1 | 4.24E-09 |
| 1 | rs6657442 | T | C | 0.779 | FXIII activity | 0.228 | 0.04 | 4.306E-09 |
| 1 | rs10801531 | G | A | 0.694 | FXIIIB | 0.209 | 0.04 | 5.201E-09 |
| 1 | rs2284664 | C | T | 0.785 | FXIII activity | 0.234 | 0.04 | 5.266E-09 |
| 1 | rs10922144 | C | T | 0.784 | FXIII activity | 0.23 | 0.04 | 5.689E-09 |
| 1 | rs1538687 | T | C | 0.692 | FXIIIB | 0.204 | 0.04 | 6.67E-09 |
| 1 | rs1576340 | G | T | 0.918 | FXIIIA | 0.436 | 0.08 | 6.679E-09 |
| 1 | rs1831281 | C | T | 0.784 | FXIII activity | 0.235 | 0.04 | 7.72E-09 |
| 1 | rs800292 | G | A | 0.768 | FXIIIB | 0.22 | 0.04 | 7.855E-09 |
| 1 | rs10737670 | A | G | 0.695 | FXIIIB | 0.205 | 0.04 | 9.844E-09 |
| 1 | rs10801530 | G | T | 0.695 | FXIIIB | 0.204 | 0.04 | 9.887E-09 |
| 1 | rs10801532 | A | G | 0.695 | FXIIIB | 0.204 | 0.04 | 9.915E-09 |
| 1 | rs12061508 | G | A | 0.79 | FXIII activity | 0.238 | 0.04 | 9.939E-09 |
| 1 | rs3855964 | G | T | 0.695 | FXIIIB | 0.204 | 0.04 | 1.01E-08 |
| 1 | rs3928857 | C | T | 0.695 | FXIIIB | 0.204 | 0.04 | 1.011E-08 |
| 1 | rs7535696 | T | C | 0.695 | FXIIIB | 0.204 | 0.04 | 1.147E-08 |
| 1 | rs10922177 | T | C | 0.838 | FXIIIB | 0.53 | 0.09 | 1.338E-08 |
| 1 | rs12069990 | C | T | 0.695 | FXIIIB | 0.202 | 0.04 | 1.344E-08 |
| 1 | rs2878557 | C | T | 0.692 | FXIIIB | 0.199 | 0.04 | 1.5E-08 |
| 1 | rs3927686 | C | T | 0.696 | FXIIIB | 0.202 | 0.04 | 1.542E-08 |
| 1 | rs12729649 | A | G | 0.695 | FXIIIB | 0.201 | 0.04 | 1.555E-08 |
| 1 | rs12069983 | C | A | 0.695 | FXIIIB | 0.201 | 0.04 | 1.555E-08 |
| 1 | rs10754187 | A | G | 0.69 | FXIIIB | 0.198 | 0.04 | 1.624E-08 |
| 1 | rs10922071 | G | A | 0.686 | FXIIIB | 0.195 | 0.04 | 1.658E-08 |
| 1 | rs10801537 | A | G | 0.686 | FXIIIB | 0.195 | 0.04 | 1.661E-08 |
| 1 | rs11807331 | T | C | 0.686 | FXIIIB | 0.195 | 0.04 | 1.661E-08 |
| 1 | rs10922177 | T | C | 0.838 | FXIII activity | 0.527 | 0.1 | 2.994E-08 |
| 1 | rs12029571 | A | G | 0.777 | FXIII activity | 0.221 | 0.04 | 3.03E-08 |
| 1 | rs7531611 | C | T | 0.684 | FXIIIB | 0.197 | 0.04 | 3.104E-08 |
| 1 | rs12065463 | T | C | 0.686 | FXIIIB | 0.191 | 0.04 | 3.718E-08 |
| 1 | rs7540032 | C | T | 0.59 | FXIII activity | 0.183 | 0.03 | 3.909E-08 |
| 1 | rs2022309 | G | T | 0.701 | D-dimer | -0.244 | 0.05 | 4.323E-08 |
| 1 | rs1769996 | T | C | 0.695 | FXIIIB | 0.196 | 0.04 | 4.44E-08 |
| 5 | rs2731672 | C | T | 0.744 | FXII Ag | 0.829 | 0.04 | 1.288E-115 |
| 5 | rs2545801 | C | T | 0.785 | FXII Ag | 0.846 | 0.04 | 2.29E-94 |
| 5 | rs1801020 | G | A | 0.798 | FXII Ag | 0.86 | 0.04 | 4.809E-88 |
| 5 | rs4976691 | G | C | 0.776 | FXII Ag | 0.812 | 0.05 | 1.376E-59 |
| 5 | rs6556312 | G | T | 0.808 | FXII Ag | 0.463 | 0.04 | 2.533E-28 |
| 5 | rs6420094 | A | G | 0.689 | FXII Ag | -0.334 | 0.04 | 1.097E-21 |
| 5 | rs3812035 | G | T | 0.691 | FXII Ag | -0.337 | 0.04 | 1.334E-21 |
| 5 | rs6862195 | G | T | 0.69 | FXII Ag | -0.333 | 0.04 | 2.613E-21 |
| 5 | rs7447593 | C | G | 0.69 | FXII Ag | -0.33 | 0.04 | 5.409E-21 |
| 5 | rs9313749 | C | A | 0.84 | FXII Ag | 0.404 | 0.04 | 5.534E-20 |
| 5 | rs10039241 | G | A | 0.865 | FXII Ag | 0.421 | 0.05 | 6.169E-19 |
| 5 | rs7710269 | G | A | 0.865 | FXII Ag | 0.421 | 0.05 | 6.592E-19 |
| 5 | rs11957608 | T | C | 0.865 | FXII Ag | 0.42 | 0.05 | 6.998E-19 |
| 5 | rs9313751 | G | A | 0.865 | FXII Ag | 0.42 | 0.05 | 7.014E-19 |
| 5 | rs7732930 | C | T | 0.865 | FXII Ag | 0.421 | 0.05 | 8.171E-19 |
| 5 | rs4976639 | C | A | 0.865 | FXII Ag | 0.421 | 0.05 | 8.18E-19 |
| 5 | rs1052432 | G | A | 0.865 | FXII Ag | 0.421 | 0.05 | 8.29E-19 |
| 5 | rs11955537 | A | G | 0.865 | FXII Ag | 0.421 | 0.05 | 8.391E-19 |
| 5 | rs2878686 | C | T | 0.865 | FXII Ag | 0.419 | 0.05 | 8.658E-19 |
| 5 | rs2431501 | A | T | 0.648 | FXII Ag | -0.296 | 0.03 | 5.322E-18 |
| 5 | rs883576 | T | C | 0.508 | FXII Ag | 0.275 | 0.03 | 2.305E-16 |
| 5 | rs2731665 | C | T | 0.508 | FXII Ag | 0.275 | 0.03 | 2.306E-16 |
| 5 | rs867755 | T | C | 0.508 | FXII Ag | 0.275 | 0.03 | 2.351E-16 |
| 5 | rs11949435 | G | C | 0.809 | FXII Ag | 0.326 | 0.04 | 3.949E-16 |
| 5 | rs12660023 | A | G | 0.809 | FXII Ag | 0.325 | 0.04 | 4.171E-16 |
| 5 | rs10077929 | T | C | 0.809 | FXII Ag | 0.325 | 0.04 | 4.225E-16 |
| 5 | rs351861 | C | A | 0.808 | FXII Ag | 0.324 | 0.04 | 6.05E-16 |
| 5 | rs4976636 | A | G | 0.808 | FXII Ag | 0.324 | 0.04 | 6.061E-16 |
| 5 | rs4976640 | A | G | 0.808 | FXII Ag | 0.324 | 0.04 | 6.094E-16 |
| 5 | rs9885210 | T | C | 0.808 | FXII Ag | 0.323 | 0.04 | 6.275E-16 |
| 5 | rs6890580 | G | A | 0.808 | FXII Ag | 0.323 | 0.04 | 6.361E-16 |
| 5 | rs2336237 | A | G | 0.808 | FXII Ag | 0.323 | 0.04 | 6.403E-16 |
| 5 | rs1363405 | C | T | 0.808 | FXII Ag | 0.323 | 0.04 | 6.426E-16 |
| 5 | rs1546363 | C | T | 0.808 | FXII Ag | 0.319 | 0.04 | 1.052E-15 |
| 5 | rs6886255 | G | A | 0.834 | FXII Ag | 0.324 | 0.04 | 3.657E-14 |
| 5 | rs10037055 | G | T | 0.835 | FXII Ag | 0.324 | 0.04 | 3.762E-14 |
| 5 | rs3812036 | C | T | 0.778 | FXII Ag | -0.321 | 0.04 | 5.691E-14 |
| 5 | rs7708314 | C | T | 0.863 | FXII Ag | 0.337 | 0.05 | 3.046E-13 |
| 5 | rs6885410 | A | C | 0.706 | FXII Ag | 0.262 | 0.04 | 3.743E-13 |
| 5 | rs6860069 | C | T | 0.703 | FXII Ag | -0.258 | 0.04 | 1.802E-12 |
| 5 | rs11749830 | G | A | 0.703 | FXII Ag | -0.258 | 0.04 | 1.819E-12 |
| 5 | rs12519145 | G | A | 0.776 | FXII Ag | 0.283 | 0.04 | 2.604E-12 |
| 5 | rs3088050 | G | A | 0.771 | FXII Ag | 0.278 | 0.04 | 3.01E-12 |
| 5 | rs4074995 | G | A | 0.741 | FXII Ag | -0.264 | 0.04 | 6.775E-12 |
| 5 | rs10866705 | A | C | 0.759 | FXII Ag | -0.261 | 0.04 | 1.029E-11 |
| 5 | rs4075958 | G | A | 0.74 | FXII Ag | -0.249 | 0.04 | 3.098E-11 |
| 5 | rs11949767 | G | A | 0.735 | FXII Ag | 0.251 | 0.04 | 3.707E-11 |
| 5 | rs3733875 | G | T | 0.89 | FXII Ag | 0.339 | 0.05 | 8.486E-11 |
| 5 | rs17078781 | G | A | 0.89 | FXII Ag | 0.337 | 0.05 | 1.084E-10 |
| 5 | rs4976682 | C | T | 0.889 | FXII Ag | 0.336 | 0.05 | 1.106E-10 |
| 5 | rs12658510 | G | A | 0.889 | FXII Ag | 0.336 | 0.05 | 1.149E-10 |
| 5 | rs6880798 | A | G | 0.889 | FXII Ag | 0.335 | 0.05 | 1.18E-10 |
| 5 | rs10072499 | T | G | 0.889 | FXII Ag | 0.335 | 0.05 | 1.222E-10 |
| 5 | rs10056655 | C | T | 0.889 | FXII Ag | 0.334 | 0.05 | 1.439E-10 |
| 5 | rs1966265 | G | A | 0.77 | FXII Ag | 0.273 | 0.04 | 2.769E-10 |
| 5 | rs478253 | C | T | 0.586 | FXII Ag | -0.206 | 0.03 | 1.01E-09 |
| 5 | rs2287694 | T | C | 0.89 | FXII Ag | 0.31 | 0.05 | 1.466E-09 |
| 5 | rs12518614 | A | G | 0.954 | vWF | -0.463 | 0.08 | 1.521E-09 |
| 5 | rs2545794 | T | C | 0.542 | FXII Ag | 0.201 | 0.03 | 1.973E-09 |
| 5 | rs628506 | A | C | 0.895 | FXII Ag | 0.321 | 0.05 | 2.678E-09 |
| 5 | rs4131289 | G | A | 0.685 | FXII Ag | -0.209 | 0.04 | 4.627E-09 |
| 5 | rs11738681 | A | G | 0.684 | FXII Ag | -0.208 | 0.04 | 5.236E-09 |
| 5 | rs11949401 | T | C | 0.684 | FXII Ag | -0.208 | 0.04 | 6.001E-09 |
| 5 | rs9313758 | T | G | 0.685 | FXII Ag | -0.208 | 0.04 | 6.163E-09 |
| 5 | rs4532376 | G | A | 0.686 | FXII Ag | -0.208 | 0.04 | 6.174E-09 |
| 5 | rs6875461 | T | C | 0.684 | FXII Ag | -0.207 | 0.04 | 6.641E-09 |
| 5 | rs6876677 | T | C | 0.684 | FXII Ag | -0.207 | 0.04 | 6.648E-09 |
| 5 | rs4976643 | A | C | 0.684 | FXII Ag | -0.207 | 0.04 | 6.695E-09 |
| 5 | rs11748912 | G | A | 0.684 | FXII Ag | -0.207 | 0.04 | 6.747E-09 |
| 5 | rs2731670 | C | A | 0.569 | FXII Ag | 0.196 | 0.03 | 1.025E-08 |
| 5 | rs2630765 | C | T | 0.55 | FXII Ag | 0.183 | 0.03 | 2.716E-08 |
| 5 | rs12654812 | G | A | 0.722 | FXII Ag | -0.211 | 0.04 | 3.002E-08 |
| 5 | rs335466 | A | C | 0.55 | FXII Ag | 0.183 | 0.03 | 3.041E-08 |
| 5 | rs337374 | A | G | 0.55 | FXII Ag | 0.183 | 0.03 | 3.041E-08 |
| 5 | rs335467 | G | A | 0.55 | FXII Ag | 0.183 | 0.03 | 3.041E-08 |
| 5 | rs335468 | A | G | 0.55 | FXII Ag | 0.183 | 0.03 | 3.041E-08 |
| 5 | rs335420 | C | T | 0.55 | FXII Ag | 0.183 | 0.03 | 3.041E-08 |
| 5 | rs3764925 | C | G | 0.55 | FXII Ag | 0.183 | 0.03 | 3.042E-08 |
| 5 | rs335434 | C | T | 0.55 | FXII Ag | 0.183 | 0.03 | 3.042E-08 |
| 5 | rs335435 | G | A | 0.55 | FXII Ag | 0.183 | 0.03 | 3.042E-08 |
| 5 | rs2731662 | G | A | 0.55 | FXII Ag | 0.183 | 0.03 | 3.042E-08 |
| 5 | rs466256 | A | C | 0.55 | FXII Ag | 0.183 | 0.03 | 3.042E-08 |
| 5 | rs465670 | T | C | 0.55 | FXII Ag | 0.183 | 0.03 | 3.042E-08 |
| 5 | rs2630764 | T | C | 0.55 | FXII Ag | 0.183 | 0.03 | 3.042E-08 |
| 5 | rs2544809 | C | T | 0.55 | FXII Ag | 0.18 | 0.03 | 4.53E-08 |
| 6 | rs5985 | C | A | 0.741 | FXIII activity | -1.077 | 0.04 | 2.586E-186 |
| 6 | rs3024321 | A | G | 0.679 | FXIII activity | -0.891 | 0.04 | 8.41E-142 |
| 6 | rs7758057 | C | G | 0.672 | FXIII activity | -0.862 | 0.04 | 2.254E-125 |
| 6 | rs1742937 | G | A | 0.667 | FXIII activity | -0.875 | 0.04 | 5.858E-120 |
| 6 | rs3024342 | T | C | 0.786 | FXIII activity | -0.855 | 0.04 | 6.543E-104 |
| 6 | rs6922262 | C | G | 0.813 | FXIII activity | -0.84 | 0.04 | 4.138E-87 |
| 6 | rs17378819 | T | C | 0.802 | FXIII activity | -0.801 | 0.04 | 2.725E-82 |
| 6 | rs1742934 | A | C | 0.627 | FXIII activity | 0.465 | 0.04 | 1.474E-38 |
| 6 | rs714408 | A | G | 0.63 | FXIII activity | 0.441 | 0.03 | 2.211E-37 |
| 6 | rs3024317 | G | A | 0.632 | FXIII activity | 0.557 | 0.04 | 4.983E-36 |
| 6 | rs3024339 | T | C | 0.942 | FXIII activity | -0.855 | 0.07 | 6.426E-36 |
| 6 | rs1475072 | T | C | 0.685 | FXIII activity | 0.397 | 0.04 | 4.987E-29 |
| 6 | rs2755416 | G | A | 0.686 | FXIII activity | 0.39 | 0.04 | 1.144E-28 |
| 6 | rs1674043 | A | T | 0.728 | FXIII activity | 0.415 | 0.04 | 1.229E-27 |
| 6 | rs1674045 | G | A | 0.729 | FXIII activity | 0.412 | 0.04 | 1.978E-27 |
| 6 | rs2755413 | C | T | 0.742 | FXIII activity | 0.411 | 0.04 | 2.209E-25 |
| 6 | rs927332 | T | C | 0.503 | FXIIIA | -0.322 | 0.03 | 1.378E-22 |
| 6 | rs11755846 | A | G | 0.941 | FXIII activity | -0.681 | 0.07 | 8.091E-22 |
| 6 | rs3863223 | G | T | 0.614 | FXIIIA | -0.301 | 0.03 | 1.047E-19 |
| 6 | rs1781790 | C | G | 0.784 | FXIII activity | 0.392 | 0.05 | 2.371E-18 |
| 6 | rs11243081 | C | T | 0.676 | FXIIIA | -0.299 | 0.04 | 5.131E-18 |
| 6 | rs4960189 | G | A | 0.676 | FXIIIA | -0.298 | 0.04 | 6.19E-18 |
| 6 | rs17142067 | A | C | 0.67 | FXIIIA | -0.299 | 0.04 | 1.24E-17 |
| 6 | rs3851517 | G | A | 0.678 | FXIIIA | -0.295 | 0.04 | 1.451E-17 |
| 6 | rs1318606 | T | C | 0.569 | FXIIIA | -0.273 | 0.03 | 4.386E-17 |
| 6 | rs12199357 | A | G | 0.679 | FXIIIA | -0.291 | 0.04 | 4.501E-17 |
| 6 | rs7766109 | A | G | 0.539 | FXIII activity | 0.267 | 0.03 | 5.613E-17 |
| 6 | rs9328347 | G | A | 0.849 | FXIII activity | -0.37 | 0.05 | 1.532E-16 |
| 6 | rs12664620 | A | G | 0.767 | FXIII activity | -0.327 | 0.04 | 2.373E-16 |
| 6 | rs1742932 | A | G | 0.698 | FXIII activity | -0.356 | 0.04 | 2.469E-16 |
| 6 | rs11243078 | T | G | 0.898 | FXIII activity | -0.412 | 0.05 | 5.346E-15 |
| 6 | rs3024370 | G | A | 0.722 | FXIII activity | -0.274 | 0.04 | 8.762E-14 |
| 6 | rs12178254 | A | G | 0.838 | FXIII activity | -0.324 | 0.05 | 1.491E-12 |
| 6 | rs11752195 | C | G | 0.587 | FXIIIA | 0.24 | 0.03 | 1.59E-12 |
| 6 | rs11754589 | A | T | 0.587 | FXIIIA | 0.239 | 0.03 | 1.745E-12 |
| 6 | rs3024371 | T | A | 0.849 | FXIII activity | -0.314 | 0.05 | 7.031E-12 |
| 6 | rs6938914 | A | T | 0.668 | FXIIIA | -0.245 | 0.04 | 9.995E-12 |
| 6 | rs3844195 | C | G | 0.667 | FXIIIA | -0.243 | 0.04 | 1.32E-11 |
| 6 | rs3863221 | A | T | 0.656 | FXIII activity | 0.233 | 0.03 | 1.385E-11 |
| 6 | rs3844196 | T | C | 0.667 | FXIIIA | -0.242 | 0.04 | 1.596E-11 |
| 6 | rs9392770 | T | C | 0.731 | FXIIIA | -0.253 | 0.04 | 2.454E-11 |
| 6 | rs6901377 | T | C | 0.731 | FXIIIA | -0.253 | 0.04 | 2.484E-11 |
| 6 | rs4959377 | T | C | 0.661 | FXIII activity | 0.226 | 0.03 | 3.754E-11 |
| 6 | rs3851514 | C | T | 0.683 | FXIII activity | 0.229 | 0.04 | 4.139E-11 |
| 6 | rs10484323 | T | C | 0.919 | FXIII activity | -0.382 | 0.06 | 7.854E-11 |
| 6 | rs9504738 | A | G | 0.921 | FXIII activity | -0.38 | 0.06 | 1.652E-10 |
| 6 | rs9504737 | G | A | 0.921 | FXIII activity | -0.38 | 0.06 | 1.687E-10 |
| 6 | rs17141966 | T | C | 0.92 | FXIII activity | -0.378 | 0.06 | 1.79E-10 |
| 6 | rs12333289 | C | T | 0.921 | FXIII activity | -0.378 | 0.06 | 2.051E-10 |
| 6 | rs1674072 | A | G | 0.934 | FXIII activity | 0.386 | 0.07 | 5.808E-09 |
| 6 | rs9405911 | G | A | 0.534 | FXIII activity | 0.195 | 0.03 | 1.23E-08 |
| 6 | rs9328354 | C | A | 0.632 | FXIIIA | 0.189 | 0.03 | 1.416E-08 |
| 6 | rs1781789 | T | C | 0.896 | FXIII activity | 0.305 | 0.05 | 1.58E-08 |
| 6 | rs11753672 | G | C | 0.633 | FXIIIA | 0.186 | 0.03 | 2.433E-08 |
| 6 | rs9504770 | C | T | 0.633 | FXIIIA | 0.186 | 0.03 | 2.433E-08 |
| 6 | rs13192814 | T | C | 0.633 | FXIIIA | 0.186 | 0.03 | 2.699E-08 |
| 6 | rs13201655 | G | T | 0.633 | FXIIIA | 0.186 | 0.03 | 2.769E-08 |
| 6 | rs13201814 | G | T | 0.633 | FXIIIA | 0.186 | 0.03 | 2.769E-08 |
| 6 | rs11243081 | C | T | 0.676 | FXIIIB | -0.187 | 0.03 | 4.359E-08 |
| 6 | rs4960189 | G | A | 0.676 | FXIIIB | -0.186 | 0.03 | 4.927E-08 |
| 9 | rs505922 | T | C | 0.68 | vWF | -0.561 | 0.04 | 4.706E-57 |
| 9 | rs529565 | T | C | 0.681 | vWF | -0.555 | 0.04 | 5.051E-56 |
| 9 | rs687621 | A | G | 0.681 | vWF | -0.555 | 0.04 | 1.152E-55 |
| 9 | rs687289 | G | A | 0.681 | vWF | -0.555 | 0.04 | 1.152E-55 |
| 9 | rs674302 | T | A | 0.681 | vWF | -0.553 | 0.04 | 2.167E-55 |
| 9 | rs514659 | A | C | 0.681 | vWF | -0.553 | 0.04 | 2.262E-55 |
| 9 | rs545971 | C | T | 0.681 | vWF | -0.553 | 0.04 | 2.266E-55 |
| 9 | rs612169 | A | G | 0.681 | vWF | -0.553 | 0.04 | 2.266E-55 |
| 9 | rs657152 | C | A | 0.655 | vWF | -0.511 | 0.03 | 2.652E-50 |
| 9 | rs644234 | T | G | 0.656 | vWF | -0.51 | 0.03 | 1.097E-49 |
| 9 | rs643434 | G | A | 0.656 | vWF | -0.51 | 0.03 | 1.097E-49 |
| 9 | rs505922 | T | C | 0.68 | FVIII Ag | -0.454 | 0.04 | 1.204E-36 |
| 9 | rs651007 | C | T | 0.81 | vWF | -0.544 | 0.04 | 4.415E-36 |
| 9 | rs687621 | A | G | 0.681 | FVIII Ag | -0.447 | 0.04 | 1.859E-35 |
| 9 | rs687289 | G | A | 0.681 | FVIII Ag | -0.447 | 0.04 | 1.859E-35 |
| 9 | rs529565 | T | C | 0.681 | FVIII Ag | -0.445 | 0.04 | 1.902E-35 |
| 9 | rs514659 | A | C | 0.681 | FVIII Ag | -0.445 | 0.04 | 2.8E-35 |
| 9 | rs545971 | C | T | 0.681 | FVIII Ag | -0.445 | 0.04 | 2.804E-35 |
| 9 | rs612169 | A | G | 0.681 | FVIII Ag | -0.445 | 0.04 | 2.804E-35 |
| 9 | rs674302 | T | A | 0.681 | FVIII Ag | -0.444 | 0.04 | 3.675E-35 |
| 9 | rs657152 | C | A | 0.655 | FVIII Ag | -0.415 | 0.04 | 1.269E-32 |
| 9 | rs644234 | T | G | 0.656 | FVIII Ag | -0.411 | 0.04 | 7.76E-32 |
| 9 | rs643434 | G | A | 0.656 | FVIII Ag | -0.411 | 0.04 | 7.76E-32 |
| 9 | rs651007 | C | T | 0.81 | FVIII Ag | -0.461 | 0.04 | 1.941E-25 |
| 9 | rs630014 | G | A | 0.521 | vWF | 0.332 | 0.03 | 5.991E-24 |
| 9 | rs633862 | T | C | 0.531 | vWF | 0.333 | 0.03 | 8.04E-24 |
| 9 | rs8176681 | T | C | 0.563 | vWF | 0.314 | 0.03 | 6.058E-22 |
| 9 | rs2073827 | G | C | 0.563 | vWF | 0.314 | 0.03 | 6.12E-22 |
| 9 | rs8176682 | C | T | 0.588 | vWF | 0.304 | 0.03 | 2.386E-20 |
| 9 | rs8176749 | C | T | 0.939 | vWF | -0.582 | 0.07 | 1.591E-17 |
| 9 | rs8176746 | G | T | 0.939 | vWF | -0.582 | 0.07 | 1.591E-17 |
| 9 | rs8176743 | C | T | 0.939 | vWF | -0.582 | 0.07 | 1.591E-17 |
| 9 | rs8176693 | C | T | 0.94 | vWF | -0.582 | 0.07 | 1.591E-17 |
| 9 | rs633862 | T | C | 0.531 | FVIII Ag | 0.286 | 0.03 | 1.827E-17 |
| 9 | rs11244079 | G | A | 0.953 | vWF | -0.61 | 0.08 | 3.287E-15 |
| 9 | rs8176749 | C | T | 0.939 | FVIII Ag | -0.536 | 0.07 | 1.189E-14 |
| 9 | rs8176746 | G | T | 0.939 | FVIII Ag | -0.536 | 0.07 | 1.189E-14 |
| 9 | rs8176743 | C | T | 0.939 | FVIII Ag | -0.536 | 0.07 | 1.189E-14 |
| 9 | rs8176693 | C | T | 0.94 | FVIII Ag | -0.536 | 0.07 | 1.189E-14 |
| 9 | rs630014 | G | A | 0.521 | FVIII Ag | 0.257 | 0.03 | 1.656E-14 |
| 9 | rs500498 | C | T | 0.527 | vWF | 0.32 | 0.04 | 5.359E-14 |
| 9 | rs8176747 | C | G | 0.932 | vWF | -0.518 | 0.07 | 2.42E-13 |
| 9 | rs9411463 | C | T | 0.95 | vWF | -0.578 | 0.08 | 3.185E-13 |
| 9 | rs8176681 | T | C | 0.563 | FVIII Ag | 0.24 | 0.03 | 4.948E-13 |
| 9 | rs2073827 | G | C | 0.563 | FVIII Ag | 0.24 | 0.03 | 5.014E-13 |
| 9 | rs495828 | G | T | 0.792 | vWF | -0.636 | 0.09 | 9.409E-13 |
| 9 | rs579459 | T | C | 0.795 | vWF | -0.645 | 0.09 | 9.852E-13 |
| 9 | rs649129 | C | T | 0.795 | vWF | -0.645 | 0.09 | 9.905E-13 |
| 9 | rs4962039 | C | T | 0.948 | vWF | -0.563 | 0.08 | 1.752E-12 |
| 9 | rs4246170 | A | G | 0.948 | vWF | -0.563 | 0.08 | 1.768E-12 |
| 9 | rs4424335 | C | G | 0.948 | vWF | -0.555 | 0.08 | 2.863E-12 |
| 9 | rs8176682 | C | T | 0.588 | FVIII Ag | 0.233 | 0.03 | 3.364E-12 |
| 9 | rs9411464 | C | T | 0.944 | vWF | -0.572 | 0.09 | 2.176E-11 |
| 9 | rs11244079 | G | A | 0.953 | FVIII Ag | -0.515 | 0.08 | 6.858E-11 |
| 9 | rs10982156 | T | A | 0.939 | FX Ag | 0.456 | 0.07 | 2.473E-10 |
| 9 | rs7853989 | G | C | 0.914 | vWF | -0.359 | 0.06 | 2.923E-10 |
| 9 | rs8176730 | T | C | 0.914 | vWF | -0.359 | 0.06 | 2.923E-10 |
| 9 | rs8176725 | G | A | 0.914 | vWF | -0.359 | 0.06 | 2.923E-10 |
| 9 | rs8176722 | C | A | 0.914 | vWF | -0.359 | 0.06 | 2.923E-10 |
| 9 | rs8176747 | C | G | 0.932 | FVIII Ag | -0.452 | 0.07 | 3.48E-10 |
| 9 | rs3124747 | G | A | 0.714 | vWF | -0.298 | 0.05 | 4.386E-10 |
| 9 | rs641959 | A | C | 0.73 | vWF | 0.215 | 0.04 | 2.148E-09 |
| 9 | rs641943 | A | G | 0.73 | vWF | 0.215 | 0.04 | 2.148E-09 |
| 9 | rs514708 | C | T | 0.73 | vWF | 0.215 | 0.04 | 2.148E-09 |
| 9 | rs672316 | T | G | 0.732 | vWF | 0.216 | 0.04 | 2.274E-09 |
| 9 | rs9411463 | C | T | 0.95 | FVIII Ag | -0.478 | 0.08 | 3.669E-09 |
| 9 | rs9411381 | T | C | 0.799 | vWF | 0.243 | 0.04 | 4.562E-09 |
| 9 | rs9411491 | T | C | 0.799 | vWF | 0.243 | 0.04 | 4.563E-09 |
| 9 | rs9411488 | G | T | 0.799 | vWF | 0.243 | 0.04 | 4.73E-09 |
| 9 | rs7853989 | G | C | 0.914 | FVIII Ag | -0.337 | 0.06 | 5.149E-09 |
| 9 | rs8176730 | T | C | 0.914 | FVIII Ag | -0.337 | 0.06 | 5.149E-09 |
| 9 | rs8176725 | G | A | 0.914 | FVIII Ag | -0.337 | 0.06 | 5.149E-09 |
| 9 | rs8176722 | C | A | 0.914 | FVIII Ag | -0.337 | 0.06 | 5.149E-09 |
| 9 | rs8176748 | C | T | 0.75 | vWF | 0.22 | 0.04 | 7.523E-09 |
| 9 | rs638756 | A | C | 0.748 | vWF | 0.216 | 0.04 | 7.861E-09 |
| 9 | rs12683493 | C | T | 0.79 | vWF | 0.231 | 0.04 | 1.304E-08 |
| 9 | rs8176717 | G | T | 0.756 | vWF | 0.21 | 0.04 | 1.384E-08 |
| 9 | rs8176740 | A | T | 0.756 | vWF | 0.21 | 0.04 | 1.452E-08 |
| 9 | rs8176732 | A | G | 0.756 | vWF | 0.21 | 0.04 | 1.452E-08 |
| 9 | rs8176728 | G | C | 0.756 | vWF | 0.21 | 0.04 | 1.452E-08 |
| 9 | rs2073825 | A | T | 0.756 | vWF | 0.21 | 0.04 | 1.452E-08 |
| 9 | rs574347 | T | C | 0.756 | vWF | 0.21 | 0.04 | 1.494E-08 |
| 9 | rs474279 | C | T | 0.757 | vWF | 0.21 | 0.04 | 1.562E-08 |
| 9 | rs8176714 | G | A | 0.756 | vWF | 0.209 | 0.04 | 1.744E-08 |
| 9 | rs517414 | G | A | 0.756 | vWF | 0.209 | 0.04 | 1.744E-08 |
| 9 | rs626035 | T | G | 0.756 | vWF | 0.208 | 0.04 | 1.819E-08 |
| 9 | rs547643 | C | T | 0.756 | vWF | 0.208 | 0.04 | 1.82E-08 |
| 9 | rs625593 | G | A | 0.756 | vWF | 0.208 | 0.04 | 1.821E-08 |
| 9 | rs549331 | C | G | 0.756 | vWF | 0.208 | 0.04 | 1.822E-08 |
| 9 | rs549446 | C | T | 0.756 | vWF | 0.208 | 0.04 | 1.823E-08 |
| 9 | rs624601 | G | A | 0.756 | vWF | 0.208 | 0.04 | 1.825E-08 |
| 9 | rs613423 | G | A | 0.756 | vWF | 0.208 | 0.04 | 1.827E-08 |
| 9 | rs579483 | T | A | 0.757 | vWF | 0.208 | 0.04 | 1.853E-08 |
| 9 | rs579622 | G | A | 0.757 | vWF | 0.208 | 0.04 | 1.855E-08 |
| 9 | rs688976 | C | A | 0.757 | vWF | 0.208 | 0.04 | 1.878E-08 |
| 9 | rs552148 | C | T | 0.757 | vWF | 0.208 | 0.04 | 1.916E-08 |
| 9 | rs568203 | C | A | 0.758 | vWF | 0.207 | 0.04 | 2.078E-08 |
| 9 | rs579459 | T | C | 0.795 | FVIII Ag | -0.519 | 0.09 | 2.28E-08 |
| 9 | rs649129 | C | T | 0.795 | FVIII Ag | -0.519 | 0.09 | 2.291E-08 |
| 9 | rs502361 | G | C | 0.759 | vWF | 0.206 | 0.04 | 2.882E-08 |
| 9 | rs500498 | C | T | 0.527 | FVIII Ag | 0.24 | 0.04 | 2.956E-08 |
| 9 | rs7025162 | C | T | 0.802 | vWF | 0.23 | 0.04 | 3.579E-08 |
| 9 | rs495828 | G | T | 0.792 | FVIII Ag | -0.504 | 0.09 | 3.718E-08 |
| 9 | rs4962039 | C | T | 0.948 | FVIII Ag | -0.447 | 0.08 | 4.584E-08 |
| 9 | rs4246170 | A | G | 0.948 | FVIII Ag | -0.446 | 0.08 | 4.619E-08 |
| 11 | rs11038993 | A | C | 0.518 | Prothrom Ag | -0.206 | 0.03 | 1.326E-10 |
| 11 | rs2306029 | C | T | 0.545 | Prothrom Ag | -0.204 | 0.03 | 2.326E-10 |
| 11 | rs4506602 | T | C | 0.512 | Prothrom Ag | -0.2 | 0.03 | 4.405E-09 |
| 13 | rs10665 | A | G | 0.878 | FVII:C | 0.847 | 0.06 | 2.369E-47 |
| 13 | rs2181540 | T | C | 0.876 | FVII:C | 0.834 | 0.06 | 1.179E-46 |
| 13 | rs6042 | C | T | 0.892 | FVII:C | 0.877 | 0.06 | 1.918E-45 |
| 13 | rs6041 | G | A | 0.892 | FVII:C | 0.877 | 0.06 | 1.927E-45 |
| 13 | rs6046 | G | A | 0.892 | FVII:C | 0.877 | 0.06 | 1.931E-45 |
| 13 | rs488703 | G | A | 0.889 | FVII:C | 0.864 | 0.06 | 3.229E-45 |
| 13 | rs1755685 | C | A | 0.892 | FVII:C | 0.888 | 0.06 | 1.856E-44 |
| 13 | rs7981123 | G | T | 0.892 | FVII:C | 0.888 | 0.06 | 2.077E-44 |
| 13 | rs561241 | T | C | 0.892 | FVII:C | 0.887 | 0.06 | 2.594E-44 |
| 13 | rs3093253 | G | A | 0.892 | FVII:C | 0.867 | 0.06 | 1.088E-43 |
| 13 | rs776905 | A | C | 0.904 | FVII:C | 0.83 | 0.07 | 9.682E-35 |
| 13 | rs556694 | T | C | 0.902 | FVII:C | 0.805 | 0.07 | 6.532E-34 |
| 13 | rs474810 | T | C | 0.898 | FVII:C | 0.811 | 0.07 | 7.865E-34 |
| 13 | rs2480946 | A | G | 0.902 | FVII:C | 0.764 | 0.07 | 2.253E-29 |
| 13 | rs776906 | A | G | 0.902 | FVII:C | 0.764 | 0.07 | 2.268E-29 |
| 13 | rs2181540 | T | C | 0.876 | FVII Ag | 0.642 | 0.07 | 1.282E-21 |
| 13 | rs488703 | G | A | 0.889 | FVII Ag | 0.673 | 0.07 | 6.725E-21 |
| 13 | rs10665 | A | G | 0.878 | FVII Ag | 0.628 | 0.07 | 1.686E-20 |
| 13 | rs6041 | G | A | 0.892 | FVII Ag | 0.673 | 0.07 | 3.263E-20 |
| 13 | rs6046 | G | A | 0.892 | FVII Ag | 0.673 | 0.07 | 3.263E-20 |
| 13 | rs6042 | C | T | 0.892 | FVII Ag | 0.674 | 0.07 | 3.278E-20 |
| 13 | rs3093253 | G | A | 0.892 | FVII Ag | 0.665 | 0.07 | 2.647E-19 |
| 13 | rs3211770 | G | A | 0.879 | FVII:C | 0.534 | 0.06 | 2.976E-19 |
| 13 | rs7327099 | T | C | 0.847 | FVII:C | 0.53 | 0.06 | 7.818E-19 |
| 13 | rs7981123 | G | T | 0.892 | FVII Ag | 0.661 | 0.08 | 1.123E-18 |
| 13 | rs1755685 | C | A | 0.892 | FVII Ag | 0.658 | 0.08 | 1.539E-18 |
| 13 | rs561241 | T | C | 0.892 | FVII Ag | 0.655 | 0.08 | 3.15E-18 |
| 13 | rs555212 | G | A | 0.768 | FVII:C | -0.367 | 0.05 | 1.071E-15 |
| 13 | rs553702 | G | A | 0.939 | FVII:C | 0.681 | 0.09 | 1.861E-14 |
| 13 | rs474810 | T | C | 0.898 | FVII Ag | 0.615 | 0.08 | 5.042E-14 |
| 13 | rs776905 | A | C | 0.904 | FVII Ag | 0.619 | 0.08 | 1.474E-13 |
| 13 | rs2480946 | A | G | 0.902 | FVII Ag | 0.587 | 0.08 | 1.98E-13 |
| 13 | rs776906 | A | G | 0.902 | FVII Ag | 0.586 | 0.08 | 2E-13 |
| 13 | rs556694 | T | C | 0.902 | FVII Ag | 0.595 | 0.08 | 2.953E-13 |
| 13 | rs3211770 | G | A | 0.879 | FVII Ag | 0.455 | 0.07 | 1.178E-11 |
| 13 | rs2146752 | A | G | 0.703 | FVII:C | 0.292 | 0.04 | 2.513E-11 |
| 13 | rs2146751 | G | A | 0.704 | FVII:C | 0.293 | 0.04 | 2.647E-11 |
| 13 | rs2026160 | A | C | 0.774 | FVII:C | 0.575 | 0.09 | 7.869E-11 |
| 13 | rs555212 | G | A | 0.768 | FVII Ag | -0.312 | 0.05 | 1.671E-09 |
| 13 | rs9533425 | G | C | 0.801 | Turb lag time | -0.308 | 0.05 | 1.904E-09 |
| 13 | rs9324220 | C | T | 0.846 | FVII:C | 0.31 | 0.05 | 1.027E-08 |
| 13 | rs2476325 | G | A | 0.846 | FVII:C | 0.306 | 0.05 | 1.576E-08 |
| 13 | rs521720 | T | C | 0.846 | FVII:C | 0.306 | 0.05 | 1.576E-08 |
| 13 | rs534298 | C | T | 0.768 | FVII:C | 0.26 | 0.05 | 2.351E-08 |
| 13 | rs2873281 | G | A | 0.847 | FVII:C | 0.301 | 0.05 | 2.885E-08 |

**Suppl Table 2**. All associations showing p<5x10^-8^ in Stage 1 (TwinsUK) are shown

**Legend to Supplementary Table 2**

Results are shown for phenotypes FXIIIA (factor XIII A subunit); FXIIIB (factor XIII B subunit); FXIII activity (factor XIII activity); FXII Ag (factor XII antigen); vWF von Willebrand factor; FVIII Ag (factor VIII antigen); FX Ag (factor X antigen); Prothrom Ag (prothrombin antigen); FVII:C (factor VII activity); FVII Ag (factor VII antigen); Turb lag time (lag time in turbidimetric assay).

**Supplementary Table 3.** Details of genotyping platforms and imputation in the Meta-Stroke studies

| Study | Source of Cases | Source of Controls | Genotyping chip  Cases | Genotyping chip  Controls | Imputation reference and (software)  Cases | Imputation reference and (software)  Controls |
| --- | --- | --- | --- | --- | --- | --- |
| ARIC | ARIC study cohort | ARIC study cohort | Affymetrix 6.0 | Affymetrix 6.0 | HapMap2 (MaCH) | HapMap2 (MaCH) |
| ASGC | Hospital cases in Perth, Adelaide, Newcastle, Gosford, Australia | Hunter Community Study | Illumina 610 | Illumina 610 | Hapmap2/3 | Hapmap2/3 |
| BRAINS | London, UK - all Caucasian | Cardiff University - all Caucasian | Illumina 660/610 | Illumina 660/610 | 1000G (MaCH) | 1000G (MaCH) |
| CEDIR | Milan study, Italy | Procardis Italian | Illumina 610 | Illumina 610 | HapMap2 (MaCH) | HapMap2 (MaCH) |
| CHS | CHS study cohort, incident strokes | CHS study cohort | Illumina 370 CNV | Illumina 370 CNV | HapMap2 (BimBam) | HapMap2 (BimBam) |
| deCODE stroke study | Icelandic DeCODE | Icelandic controls | 317/370 Illumina | 317/370 Illumina | HapMap2 (SNPTEST) | HapMap2 (SNPTEST) |
| FHS | Framingham Study Original and Offspring cohort subjects | Framingham Study Original and Offspring cohort subjects | Affymetrix 550K (500K+50K Human gene focused panel) | Affymetrix 550K (500K+50K Human gene focused panel) | HapMap2 (MaCH) | HapMap2 (MaCH) |
| GASROS | European ancestry; from Massachusetts General Hospital | European ancestry; from Massachusetts General Hospital | Affymetrix 6.0 | Affymetrix 6.0 | HapMap3 (PLINK) | HapMap3 (PLINK) |
| GEOS | Baltimore-Washington metropolitan area | Baltimore-Washington metropolitan area | Illumina HumanOmni1-Quad_v1-0_B | Illumina HumanOmni1-Quad_v1-0_B | NA | NA |
| HPS | HPS study | HPS study | Illumina 610 | Illumina 610 | HapMap2 (MaCH) | HapMap2 (MaCH) |
| HVH | Group Health Cooperative | Group Health Cooperative | Illumina 370 CNV | Illumina 370 CNV | HapMap2 (BimBam) | HapMap2 (BimBam) |
| ISGS | Ischaemic Stroke Genetics Study | includes SWISS | Illumina 610 | Illumina 610 | 1000G (MaCH) | 1000G (MaCH) |
| Rotterdam | Rotterdam Study | Rotterdam Study | Illumina HumanHap 550 Duo Beadchip | Illumina HumanHap 550 Duo Beadchip | HapMap 2 (MaCH) | HapMap 2 (MaCH) |

**Legend to Supplementary Table 3`**

NA represents not available

1000G represents 1000 Genomes Project reference database
